# Five autism-associated transcriptional regulators target shared loci proximal to brain-expressed genes

**DOI:** 10.1016/j.celrep.2024.114329

**Published:** 2024-06-07

**Authors:** Siavash Fazel Darbandi, Joon-Yong An, Kenneth Lim, Nicholas F. Page, Lindsay Liang, David M. Young, Athena R. Ypsilanti, Matthew W. State, Alex S. Nord, Stephan J. Sanders, John L.R. Rubenstein

**Affiliations:** 1 Nina Ireland Laboratory of Developmental Neurobiology, University of California, San Francisco, San Francisco, CA 94143, USA; 2 Department of Psychiatry and Weill Institute for Neurosciences, University of California, San Francisco, San Francisco, CA 94143, USA; 3 School of Biosystem and Biomedical Science, College of Health Science, Korea University, Seoul, South Korea; 4 BK21FOUR R&E Center for Learning Health Systems, Korea University, Seoul, South Korea; 5 Department of Neurobiology, Physiology, and Behavior and Department of Psychiatry and Behavioral Sciences, Center for Neuroscience, University of California, Davis, Davis, CA 95618, USA; 6 Bakar Computational Health Sciences Institute, University of California, San Francisco, San Francisco, CA 94143, USA; 7 Institute for Human Genetics, University of California San Francisco, San Francisco, CA 94143, USA; 8 Institute for Developmental and Regenerative Medicine, Old Road Campus, Roosevelt Dr., Headington, Oxford OX3 7TY, UK; 9 These authors contributed equally; 10 Lead contact

## Abstract

Many autism spectrum disorder (ASD)-associated genes act as transcriptional regulators (TRs). Chromatin immunoprecipitation sequencing (ChIP-seq) was used to identify the regulatory targets of ARID1B, BCL11A, FOXP1, TBR1, and TCF7L2, ASD-associated TRs in the developing human and mouse cortex. These TRs shared substantial overlap in the binding sites, especially within open chromatin. The overlap within a promoter region, 1–2,000 bp upstream of the transcription start site, was highly predictive of brain-expressed genes. This signature was observed in 96 out of 102 ASD-associated genes. *In vitro* CRISPRi against ARID1B and TBR1 delineated downstream convergent biology in mouse cortical cultures. After 8 days, NeuN+ and CALB+ cells were decreased, GFAP+ cells were increased, and transcriptomic signatures correlated with the postmortem brain samples from individuals with ASD. We suggest that functional convergence across five ASD-associated TRs leads to shared neurodevelopmental outcomes of haploinsufficient disruption.

## In brief

Using NGS approaches, Fazel Darbandi et al. demonstrate that ASD-associated TRs in the developing cortex share substantial overlap in their binding sites. *In vitro* CRISPRi delineated downstream convergent biology that correlates with the postmortem brain samples from individuals with ASD. We suggest that functional convergence of ASD-associated TRs leads to shared neurodevelopmental disorders.

## Graphical Abstract

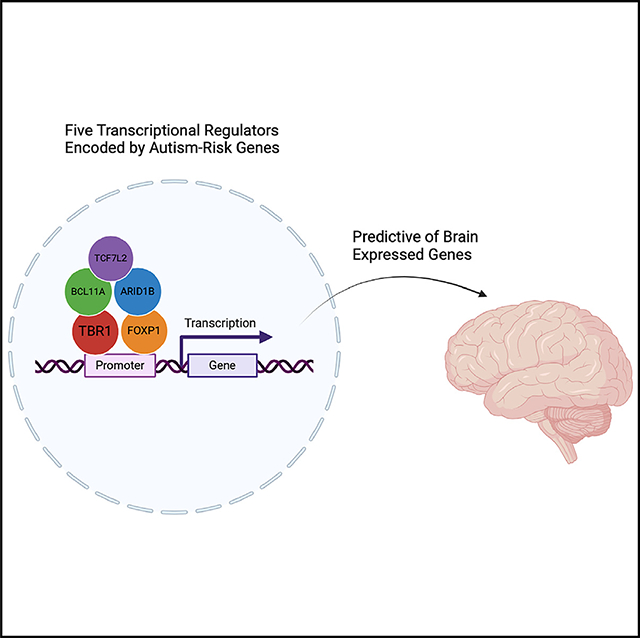

## INTRODUCTION

Autism spectrum disorder (ASD) is a common and highly heritable neurodevelopmental disorder.^1^ To date, over a hundred genes have been associated with ASD, mostly through the detection of rare loss-of-function variants that disrupt the function of one of the two copies of a gene.^2,3^ However, the mechanism by which disruption of these genes leads to ASD symptoms remains elusive. Analysis of patterns of gene expression for these ASD-associated genes in the developing human brain has implicated excitatory and inhibitory cortical and striatal neurons.^2–6^ Orthogonal analysis of the postmortem brain in ASD cases identifies down-regulated gene expression modules, which are enriched for both neuronal marker genes and ASD-associated genes, and up-regulated gene expression modules enriched for non-neuronal marker genes but not ASD-associated genes.^7–9^ Mouse experiments observed similar transcriptomic patterns as a consequence of disrupting multiple ASD-associated genes, with some gene expression profiles overlapping those seen in the postmortem ASD brain.^10^ These results suggest that convergent pathology, captured by high-dimensional transcriptomic datasets, may underlie the shared phenotypic consequences across multiple ASD-associated genes. This, in turn, raises the question of how disrupting multiple genes with heterogeneous and pleiotropic functions can yield similar transcriptomic and phenotypic outcomes.

The majority of ASD-associated genes encode proteins that act as transcriptional regulators (TRs), influencing the expression of other genes; these include transcription factors (e.g., *TBR1*, *FOXP1*), histone modifiers (e.g., *KMT5B*), and chromatin remodelers (e.g., *CHD8*).^2^ The genomic targets of these regulatory genes and the transcriptomic consequences of their disruption remain largely uncharacterized, as do their functional relationship to other ASD-associated genes. Identifying these genomic targets could reveal convergent gene regulatory networks and predict downstream neurobiology to account for the shared autistic phenotype; they could also provide an orthogonal approach to distinguish the cell types, brain regions, and developmental stages involved. Analyses of the genomic targets of individual genes support this possibility, for example, targets of CHD8, POGZ, and TBR1 are enriched for ASD-associated genes^11–14^; however, the identification of shared targets is complicated by heterogeneous protocols, species, cells/tissues, and developmental stages.

**Figure 1.**
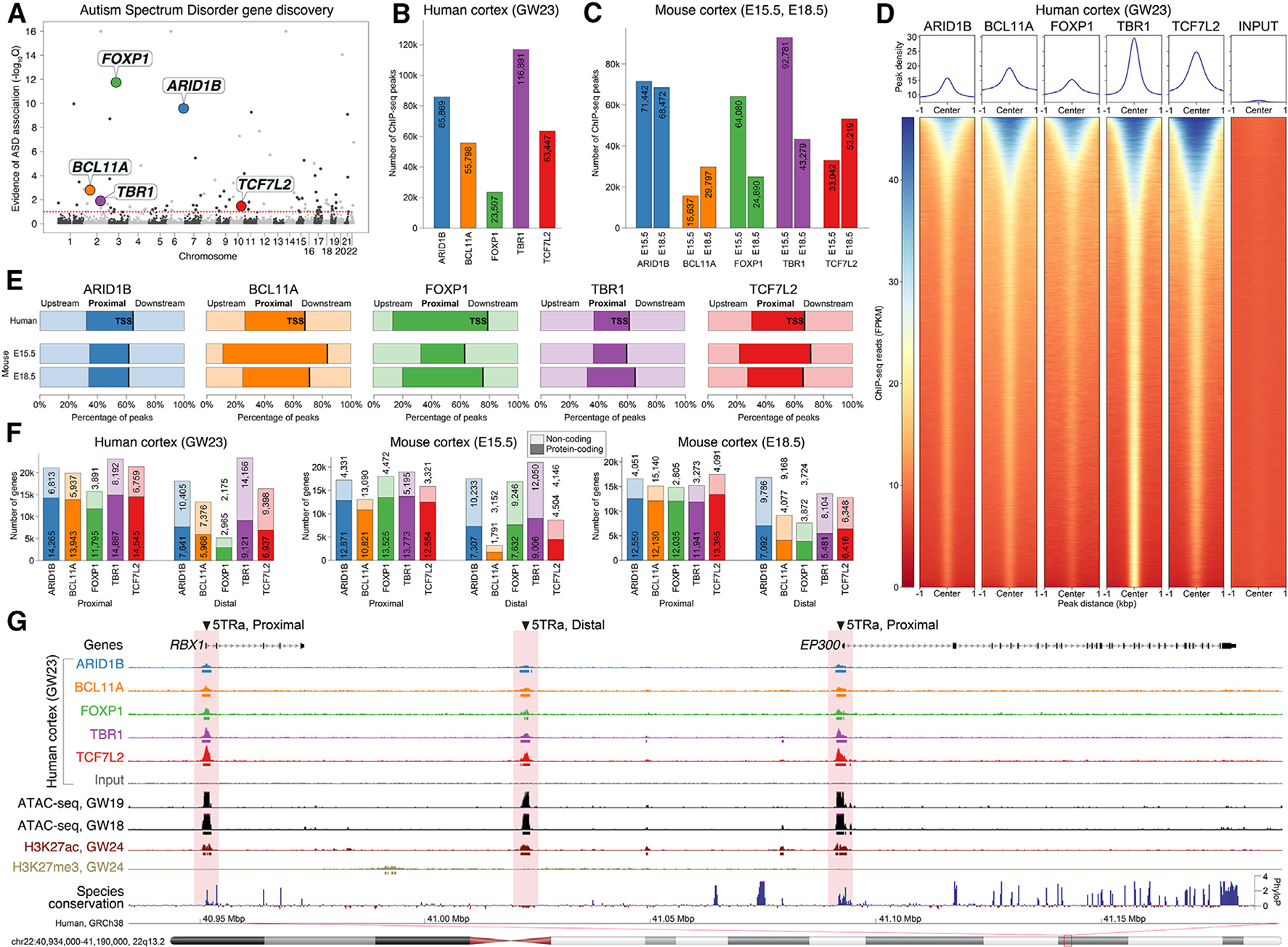
ChIP-seq peaks identified in five ASD-associated TRs (A) Evidence of ASD association for five DNA-binding TRs from exome sequencing.^2^ (B and C) ChIP-seq peaks were identified in (B) human cortex at gestation week 23 (GW23) and (C) mouse cortex (embryonic days 15.5 and 18.5). (D) Read counts around ChIP-seq peaks for human cortex. The union of peaks across the five TRs is shown in the same order (y axis) for all six datasets (additional datasets shown in Figure S1). (E) ChIP-seq peaks for all five transcription factors are enriched for promoter regions ≤2,000 bp proximal to the transcription start site (TSS). (F) ChIP-seq peaks are found proximal to the TSS of 10,663 to 14,874 protein-coding genes across species and assay. (G) A representative example of TR peaks in human prefrontal cortex proximal and distal to two genes are shown alongside peaks from ATAC-seq in GW18 andGW19 human prefrontal cortex and histone ChIP-seq data (H3K27ac, H3K27me3) in GW24 human prefrontal cortex (compared to an ATAC-seq only region). 5TRa, locus with binding by all five ASD-associated TRs (5TRs) and an ATAC-seq peak (a); E15.5/E18.5, embryonic days 15.5/18.5; GW, gestation week. See also Figures S1 and S2.

To assess the extent of shared regulatory targets across ASD-associated genes, we selected five TRs for further analysis, based on strong evidence for ASD association (Figure 1A), expression during cortical development, evidence of direct binding to DNA, and the availability of reliable antibodies. *ARID1B* and *BCL11A* are both DNA-binding subunits of the BAF (SWI/SWF) chromatin remodeling complex expressed across multiple tissues,^15,16^ though cortical *BCL11A* expression appears restricted to neurons,^17,18^ Both *FOXP1*, a winged-helix TR, and *TBR1*, a T-Box TR, are expressed highly in neurons of the developing cortex, and *FOXP1* is also expressed in neurons of the developing striatum.^17–20^ *TCF7L2* is an HMG-Box TR expressed in the cortical and subcortical progenitors^21^ and thalamic excitatory neurons.^22^ We observed substantial overlap in the genomic targets of all five TRs in the developing human and mouse cortex, especially in proximity to genes highly expressed in the cortex, including most ASD-associated genes. These shared genomic targets are proximal to genes critical to brain development and function and thus suggest a mechanism for similar phenotypic consequences for mutations in the diverse set of ASD genes.

## RESULTS

### Identifying regulatory targets of five ASD-associated TRs in developing human and mouse neocortex

We generated chromatin immunoprecipitation sequencing (ChIP-seq) data for these five ASD-associated TRs from human frontal neocortex at gestational week 23 (GW23) and mouse neocortex at embryonic day (E) 15.5 and E18.5 (Table S1; NCBI’s Gene Expression Omnibus [GEO] under GEO: GSE248876).

Peak counts ranged from 23,507 to 116,891 (Figures 1B and 1C), were distinct from reads in input and blocking peptide controls (Figures 1D and S1), were consistent across biological replicates and with previously published data^13^ (Figure S2), and showed substantial conservation across species. Four of the TRs have known motifs (BCL11A, FOXP1, TBR1, and TCF7L2), which were enriched in the corresponding ChIP-seq peaks compared to the scrambled sequence (Figure S2E). In contrast, previously published transposase-accessible chromatin sequencing (ATAC-seq) peaks^23^ were present at numerous loci without TR peaks (Figures S1 and S5). A high proportion of ChIP-seq loci were proximal peaks, defined as overlapping the promoter region, mapping 0–2,000 bp upstream of any transcription start site (TSS), compared to distal peaks, defined as peaks not overlapping promoters (Figure 1E). Despite considerable variation in peak counts (Figures 1B and 1C), the number of protein-coding genes with a proximal peak was similar between TRs, species, and developmental stages, ranging from 10,663 to 14,874 genes (Figure 1F).

### ASD-associated TRs converge on a common set of targets

**Figure 2.**
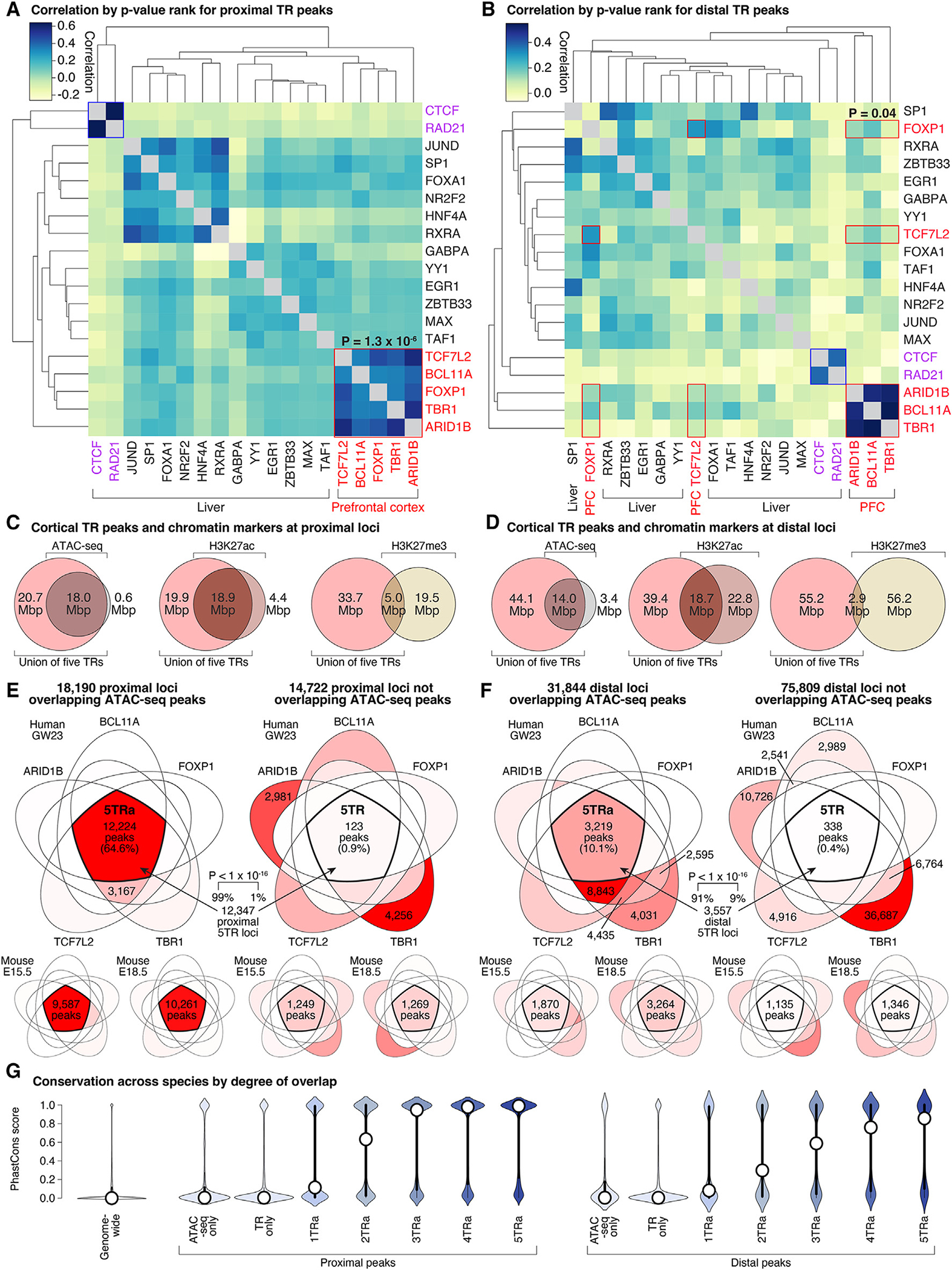
Overlap between ChIP-seq peaks from five ASD-associated TRs (A) Top 10,000 proximal ChIP-seq peaks ranked by *p* value from 14 TRs in adult human liver (ENCODE, Table S2) and five ASD-associated TRs in fetal human cortex (Table S1) to assess intersection (Figure S3A) and correlation of peak ranks. (B) Equivalent plot for 8,000 distal peaks. (C) Overlap between proximal ChIP-seq peaks for five ASD-associated TRs and three epigenetic markers in human fetal cortex. (D) Equivalent overlaps for distal peaks. (E and F) Overlap between proximal ChIP-seq peaks for the five ASD-associated TRs in developing human cortex overlapping (top left) or not overlapping (top right) with ATAC-seq peaks. Color gradient represents the percentage of peaks in each section, with red being the highest percentage and white being 0%; peak counts are given for the intersection of all five TRs and sections with greater than 1,500 peaks. Equivalent plots are shown for E15.5 mouse cortex and E18.5 mouse cortex (bottom) and (F) distal peaks. (G) PhastCons scores for conservation across 100 vertebrate species are shown genome wide (left), for loci with ATAC-seq peaks but no ChIP-seq TR peaks or ChIP-seq TR peaks by no ATAC-seq peaks, and for ATAC-seq peaks intersecting with one to five ChIP-seq TR peaks (1TRa–5TRa). 5TRa, peak with all five ASD-associated transcription factors (5TRs) and ATAC-seq (a); GW23, gestational week 23; E15.5/E18.5, embryonic days 15.5/18.5. Statistical analyses: (A and B) Wilcoxon test; (E) permutation test. See also Figures S3–S5.

The ChIP-seq peaks for the five ASD-associated TRs frequently targeted the same genomic loci in developing human cortex (Figures 1D and 1G), suggesting shared regulatory networks and/or protein complexes.^24^ To assess the expected degree of overlap between TRs in heterogeneous tissues, we reprocessed ENCODE ChIP-seq data for 14 TRs in the adult human liver through our analysis pipeline (Table S2). We used *p* values to identify the top 10,000 proximal peaks for each TR and assessed the intersection and correlation by *p* value rank for all combinations of TRs. The ASD-associated genes showed a greater degree of both correlation (0.42 Spearman’s rho in cortex vs. 0.07 in liver, *p* = 1.3 × 10^−6^, Wilcoxon; Figure 2A) and intersection (83.8% of intersecting peaks in cortex vs. 59.2% in liver, *p* = 2.7 × 10^−7^, Wilcoxon; Figure S3A) than TRs in the liver, except for CTCF and RAD21 (components of the chromatin looping complex^25^). Performing this analysis for 8,000 distal peaks also showed high overlap, especially between ARID1B, BCL11A, and TBR1 (0.26 Spearman’s rho in cortex vs. 0.13 in liver, *p* = 0.04 for correlation, Figure 2B; 46.1% of intersecting peaks in cortex vs. 36.0% in liver, *p* = 0.37; Figure S3B).

To further understand the implications of these overlapping TR peaks in developing human cortex, we considered the overlap with open chromatin regions, detected by the ATAC-seq in GW18/19 human cortex,^23^ and H3K27ac and H3K27me3 histone modifications detected by ChIP-seq in GW24 human cortex (Figures 2C and 2D). We observed a substantial overlap between the proximal ASD-associated TR peaks and ATAC-seq (55.5% of TR peaks and 46.5% of nucleotides covered by TR peaks) and H3K27ac peaks (53.4% of peaks and 48.7% of nucleotides), both of which are associated with active transcription. In contrast, overlap with H3K27me3, a marker of gene repression, was minimal (16.5% of peaks and 13.0% of nucleotides). A similar pattern was observed in the developing human cortex for distal peaks (Figure 2D). Thus, the five ASD-associated TRs predominantly bind to proximal and distal loci with epigenetic states indicative of active transcription.

We next considered whether overlaps between ChIP-seq peaks of multiple ASD-associated TRs (Figures 1G, 2A, and 2B) occurred within the ATAC-seq-defined open chromatin regions or not (Figures 2C, 2D, S4, and S5). In the developing human cortex, we identified 32,962 independent proximal loci targeted by one or more ASD-associated TRs, split approximately in half between those with concurrent ATAC-seq peaks (18,190, 55.2%) and those without (14,772, 44.8%). Of the 32,962 proximal loci, 12,347 (37.5%) are targeted by all five ASD-associated TRs (5TRs), and remarkably, almost all of these have concurrent ATAC-seq peaks (12,224, 99.0% of 5TR proximal peaks, 64.6% of all proximal peaks; *p* < 1 × 10^−10^, permutation testing to account for size differences, Figure 2E). In contrast, of the 10,736 proximal loci targeted by a single ASD-associated TR, only 882 (8.2%) had concurrent ATAC-seq peaks. Similar enrichment for overlapping ASD-associated TR ChIP-seq peaks within ATAC-seq peaks was observed for mouse cortical data at both E15.5 and E18.5 (Figure 2E) and for developing human cortex data within H3K27ac peaks (Figures S3C and S3D).

Enrichment for overlapping ASD-associated TR ChIP-seq peaks was also observed within ATAC-seq peaks for distal peaks (Figure 2F). The 107,653 independent distal peaks included 31,844 (29.6%) with concurrent ATAC-seq peaks and 75,809 (70.4%) without. Out of 3,557 distal loci targeted by all five ASD-associated TRs, 3,219 (90.5%) have concurrent ATAC-seq peaks (*p* < 1 × 10^−10^, permutation testing, Figure 2F), while most of the 62,474 distal loci targeted by a single ASD-associated TR occur outside of ATAC-seq-marked open chromatin regions (55,911, 89.5%). There were 6,063 distal loci identified by ATAC-seq without any of the five ASD-associated TRs. As with proximal loci, similar patterns are seen in mouse cortical data (Figure 2F) and for H3K27ac peaks in the developing human cortex (Figures S3E and S3F).

### Open chromatin regions targeted by all five ASD-associated TRs are highly conserved

Given the high degree of overlap between ATAC-seq peaks and ChIP-seq peaks from all five ASD-associated TRs, hereafter referred to as “5TRa,” we sought to characterize these regions in depth. The 12,224 proximal 5TRa loci in the developing human cortex span 26.2 Mbp and are upstream of the TSS of 11,695 protein-coding transcripts and 4,739 non-coding transcripts (Table S3). ChIP-seq peaks for each TR contributing to the 5TRa regions were called with higher confidence, based on lower p values, than peaks outside the 5TRa regions (Figure S3G). Most of these proximal loci are highly conserved across species, with 8,233 (67.4%) including a region with a max PhastCons score above 0.5 (Figure 2G). Moreover, 8,867 (72.5%) overlap with a 5TRa peak in mouse E15.5 cortex, and 8,639 (70.7%) overlap with a 5TRa proximal loci in mouse E18.5 cortex.

The 3,219 distal 5TRa regions from the developing human cortex span 5.7 Mbp. Based on the nearest TSS, these loci are related to 850 protein-coding transcripts and 1,932 non-coding transcripts (Table S3). As seen for proximal loci, the distal 5TRa loci have lower *p* values than those outside of 5TRa loci (Figure S3H). Most are highly conserved across species, with 1,880 (58.4%) loci including a region with a max PhastCons score above 0.5 (Figure 2G). Furthermore, 652 (20.3%)/548 (17.0%) overlap 5TRa distal loci in mouse E15.5/E18.5 cortex.

### ASD-associated TR-bound loci are enriched for motifs of TR genes associated with other neurodevelopmental and psychiatric disorders

**Figure 3.**
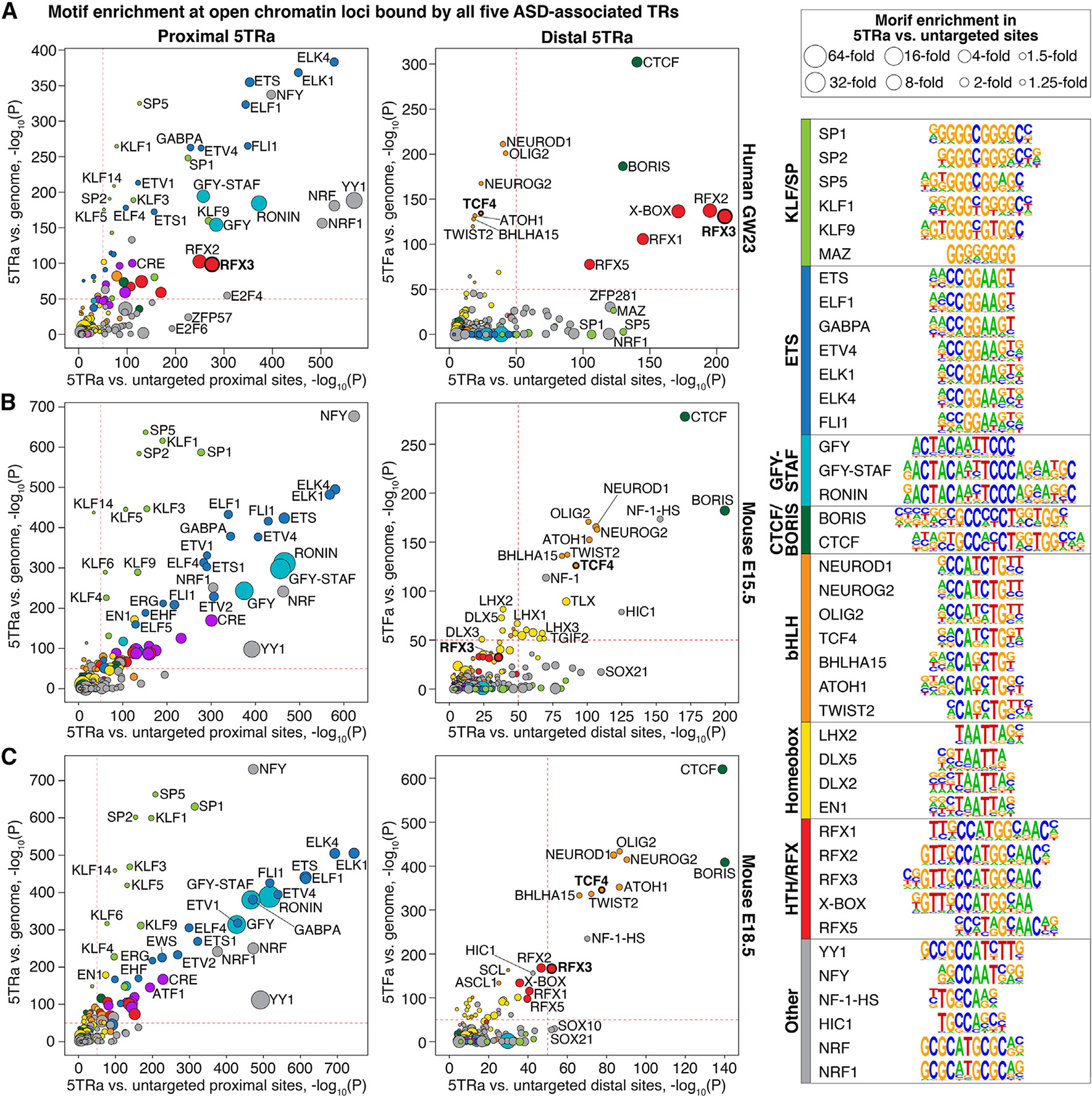
Motif enrichment in regions targeted by ASD-associated TRs (A) Results of HOMER known motif enrichment (Table S4) for proximal (left) and distal (right) loci bound by all five ASD-associated TRs and ATAC-seq (5TRa) against genomic background (y axis) and against untargeted proximal/distal sites (x axis, supplemental information). Groups of related motifs are shown by color (image on right) and motifs for ASD-associated genes (e.g., *RFX3*) are in bold font. Dashed red lines are included at *p* = 1 × 10^−50^ to aid comparisons across images. (B and C) These analyses are repeated for mouse 5TRa loci at (B) E15.5 and (C) E18.5. bHLH, basic-helix-loop-helix; CTCF, CCCTC-binding factor; ETS, E-twenty-six transformation-specific; GFY, general factor Y; HIC1, HIC ZBTB transcriptional repressor 1; HTH, helix-turn-helix; KLF, Krüppel-like family; NFY, nuclear transcription factor Y; RFX, regulatory factor binding to the X-box; SP, specificity protein; STAF, selenocysteine tRNA gene transcription-activating factor (ZNF143). Statistical analyses: (A–C) HOMER binomial enrichment.

Since the five ASD-associated TRs have DNA-binding domains, we used HOMER to assess whether DNA sequence motifs were enriched in the 5TRa proximal and distal loci against both a genomic background and representative proximal/distal backgrounds (Figure 3). Similar results were obtained using 5TRa loci from the developing human and mouse cortex. Substantial enrichment was observed for motifs related to promoter-enhancer loops, including ZNF143/Staf and THAP11/Ronin in proximal elements and CTCF and CTCFL/Boris in distal elements (Figure 3). Proximal 5TRa loci were also enriched for the ETS, KLF/SP, YY1, NKRF/Nrf, and NFY motif groups, while distal loci were enriched for the HTH/RFX and the basic-helix-loop-helix (bHLH) motif groups. Many of the genes in these 5TRa-enriched motif groups are associated with neurodevelopmental and psychiatric disorders (10 out of 182 genes; 3.4-fold enrichment; *X*^2^ [1, *N* = 19,654] = 15.9, *p* = 7 × 10^−5^; Table S4), including ASD^2^: *RFX3* (HTH/RFX), *TCF4* (bHLH), and *NCOA1* (bHLH); neurodevelopmental delay^26^: *CTCF* (CTCF/BORIS), *YY1* (YY1), *ERF* (ETS), *KLF7* (KLF/SP), *TFE3* (bHLH), and *MYCN* (bHLH); and schizophrenia^27^: *SP4* (KLF/SP).

### ASD-associated TRs are proximal to ASD-associated genes and brain-expressed genes

**Figure 4.**
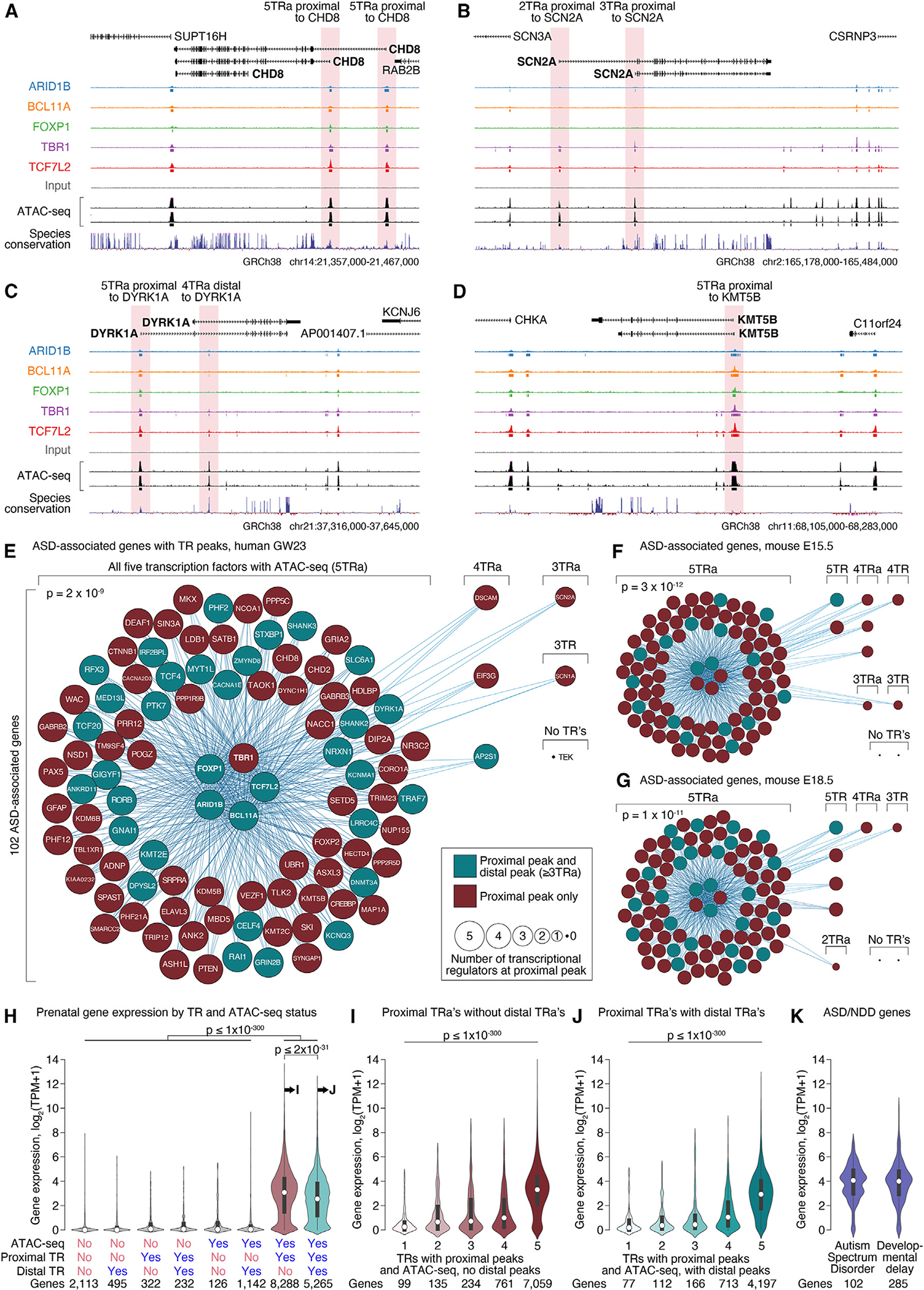
Enrichment of overlapping TR peaks at ASD-associated genes (A–D) TR peaks around the ASD-associated genes *CHD8, SCN2A, DYRK1A,* and *KMT5B*. (E) Network plot showing whether ChIP-seq peaks for the five ASD-associated TRs (central circles/nodes) and ATAC-seq are detected proximal to the other 97 ASD-associated genes (peripheral circles/nodes, Table S5). Genes that also have a nearby distal peak that includes ARID1B, BCL11A, TBR1, and ATAC-seq (Figures 2C and 2H) are shown in teal, while those without such a peak are in brown. (F and G) Equivalent plots are shown for the same genes for the mouse data at (F) E15.5 and (G) E18.5. (H) Median gene expression in the fetal human prefrontal cortex is represented for protein-coding genes binned by the presence or absence of at least one TR proximally or distally with or without ATAC-seq. (I and J) Median gene expression in the fetal human prefrontal cortex is shown for all cortex-expressed protein-coding genes, binned by the number of ASD-associated TRs bound proximally (I) in the absence of a distal TR and (J) in the presence of a distal TR. (K) Equivalent expression of genes associated with ASD or developmental delay. 5TRa, peak with all five ASD-associated TRs and ATAC-seq; TPM, transcripts per million (a). Statistical analyses: (E–G) chi-squared; (H–J) logistic regression. See also Figure S6.

Prior analyses have described enrichment of ASD-associated genes in proximity to the binding sites of individual ASD-associated TRs.^11–14^ We observed a similar result with proximal 5TRa loci from the developing human cortex upstream of 96 out of 102 ASD-associated genes^28^ (94.1% of ASD genes vs. 65.7% of non-ASD genes; *X*^2^ [1, *N* = 17,484 all autosomal protein-coding genes] = 36.2, *p* = 2 × 10^−9^; Figure 4; Table S5). For the remaining six ASD-associated genes, five had overlapping peaks from at least three of the ASD-associated TRs proximal to their TSS. Equivalent results were also observed using data from a mouse at E15.5 and E18.5 (Figures 4F and 4G). Assessing ASD enrichment by permutation test, accounting for gene cDNA length (a predictor of ASD gene discovery), yielded a similar result (*p* = 5 × 10^−4^).

We next considered whether the presence of an ATAC-seq peak, or a proximal ASD-associated TR peak, predicted gene expression levels in the developing (GW12–40) human prefrontal cortex.^6^ Remarkably, most genes with proximal ATAC-seq peaks and at least one ASD-associated TR peak were robustly expressed (log_2_(TPM+1) ≥ 1), whereas almost all genes without both a proximal ATAC-seq and at least one ASD-associated TR peak were weakly expressed (log_2_(TPM+1) < 1, Figure 4H). For peaks with both a proximal ATAC-seq peak and an ASD-associated TR peak, the number of ASD-associated TRs was highly predictive of the level of gene expression; this relationship was non-linear on a logarithmic scale of expression, with much higher expression in the presence of an ATAC-seq peak and all five ASD-associated TRs than four or fewer (Figures 4I, 4J, and S9). Genes associated with ASD are highly brain expressed (median log_2_(TPM+1) = 4.0, Figure 4K), and correcting for gene expression in the brain accounts for the extent of their enrichment in 5TRa loci, suggesting that the binding of multiple ASD-associated TRs proximal to ASD genes may contribute to their high brain expression.

### Genes with ASD-associated TR peaks have higher expression in the fetal brain

Target genes of distal loci were identified using a nearest TSS approach, including all protein-coding and non-coding transcripts. Given the overlap between ARID1B, BCL11A, and TBR1 at distal ATAC-seq peaks (Figure 2), we assessed the number of ASD-associated genes with distal loci in the developing human cortex containing at least these factors (≥3TRa). The 102 ASD-associated genes^28^ were also enriched for these distal peaks (35.3% of ASD genes vs. 12.1% of non-ASD genes; *X*^2^ [1, *N* = 17,484 all autosomal protein-coding genes] = 50.5, *p* = 1 × 10^−12^; Figure 4; Table S5). However, the presence of a distal locus was associated with slightly lower gene expression in the developing human prefrontal cortex (Figures 4H and 4J).

The enrichment we observed for GFY-STAF/ZNF143/THAP11 and CTCF/BORIS/CTFL motifs at 5TRa loci suggests that the 5TRa loci participate in chromosomal looping. Consistent with this idea, we identified potential interactions between ≥3TRa distal peaks and 77 of the 102 (75.5%) ASD-associated genes (Table S5) using the activity-by-contact (ABC) approach^29^ with chromatin accessibility and Hi-C data from the developing human brain.^23,30^ Similarly, a distal ≥3TRa peak was detected within 100 kb of 70 ASD-associated genes (68.6%) and within 500 kb for 96 (94.1%).

To better understand the function of these distal peaks, we considered the overlap with experimentally validated enhancer loci defined by VISTA.^31^ Of the 998 VISTA human elements with activity in E11.5 mice, 53 overlap with a 5TRa region, and 140 overlap with a ≥3TRa region that includes ARID1B, BCL11A, and TBR1 (Figure 2B). As expected for a dataset derived from cortex, we observed enrichment for VISTA elements that are active in the telencephalon (63 regions, odds ratio = 2.17, *p* = 5 × 10^−5^), including elements specific to both the pallium and subpallium in the E11.5 mouse (Figure S6;Table S6). Two of these VISTA-positive regions also showed evidence of an interaction with ASD genes through the ABC data: hs399 with *BCL11A* and hs416 with *ARID1B* (Table S6).

**Figure 5.**
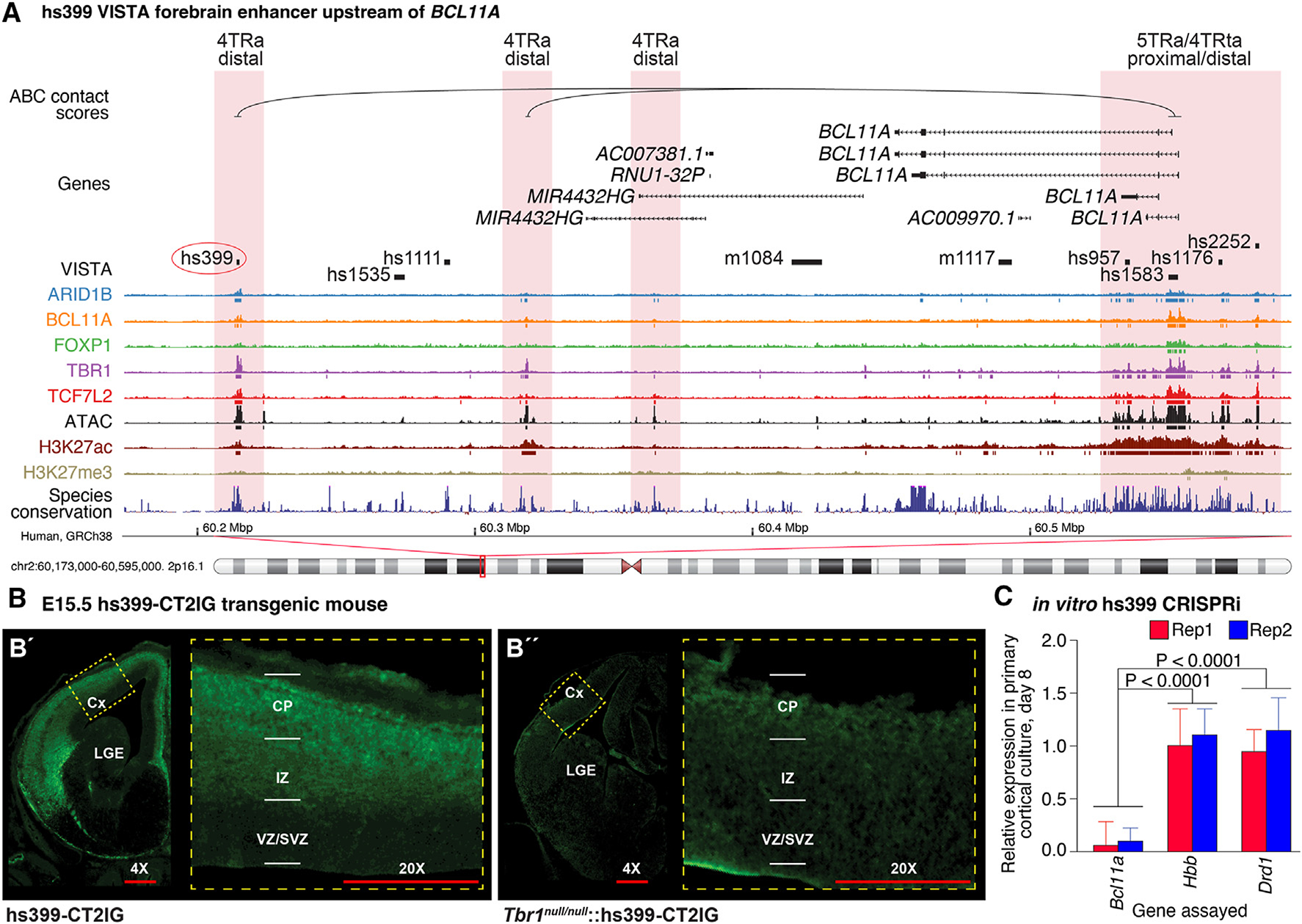
hs399 distal locus regulates *Bcl11a* expression in the developing mouse cortex (A) Four TRs encoded by ASD-associated genes (ARID1B, BCL11A, TBR1, and TCF7L2) bind to VISTA element hs399 in the human prefrontal cortex at GW23, overlapping with ATAC-seq and H3K27ac ChIP-seq peaks. ABC data show a relationship with the *BCL11A* TSS 340,000 bp downstream. (B) hs399 is active in the cortical plate and intermediate zone of an hs399-CT2IG enhancer transgenic mouse at E13.5. TBR1 promotes the activity of the hs399 putative regulatory element, as the hs399 activity (GFP expression) is reduced in *Tbr1*^*null/null*^. Anti-GFP immunostaining is in green. (C) qPCR analysis of *in vitro* CRISPRi guide RNA directed against hs399 enhancer in mouse primary neocortical cultures 8 days post-transduction. CRISPRi directed to the hs399 locus decreased *Bcl11a* expression but did not impact *Hbb* and *Drd1* expression. Statistical analysis: two-tailed t test with Tukey correction was used for pairwise comparisons. Error bars represent standard error of the mean of two biological replicates. Cx, cortex; CP, cortical plate; IZ, intermediate zone; LGE, lateral ganglionic eminence; SVZ, subventricular zone; VZ, ventricular zone. 4× and 20× refer to relative magnification. See also Figure S6.

Next, we directly tested whether the hs399 VISTA region is regulated by TBR1. hs399 is ~340 kbp downstream of the *BCL11A* TSS and is bound by ARID1B, BCL11A, TBR1, and TCF7L2 in an open chromatin region (4TRa) (Figure 5A). ABC analysis showed a potential interaction between this locus and the *BCL11A* TSS (Figure 5A). We generated a stable hs399 enhancer transgenic mouse (hs399-CT2IG) that expresses GFP and Cre^ERT2^.^32^ Immunohistochemistry shows that this regulatory element is active in the prenatal and post-natal developing cortex (Figure 5B; data not shown). However, in a constitutive *Tbr1*^*nul/null*^ background, hs399 enhancer activity is substantially reduced (Figure 5B”). Furthermore, CRISPRi targeted to the hs399 element in cultured neonatal mouse cortical neurons resulted in an ~10-fold decrease in *BCL11A* RNA expression (Figure 5C).

### ASD-associated TRs target genes whose expression is enriched in cortical progenitors and neurons

**Figure 6.**
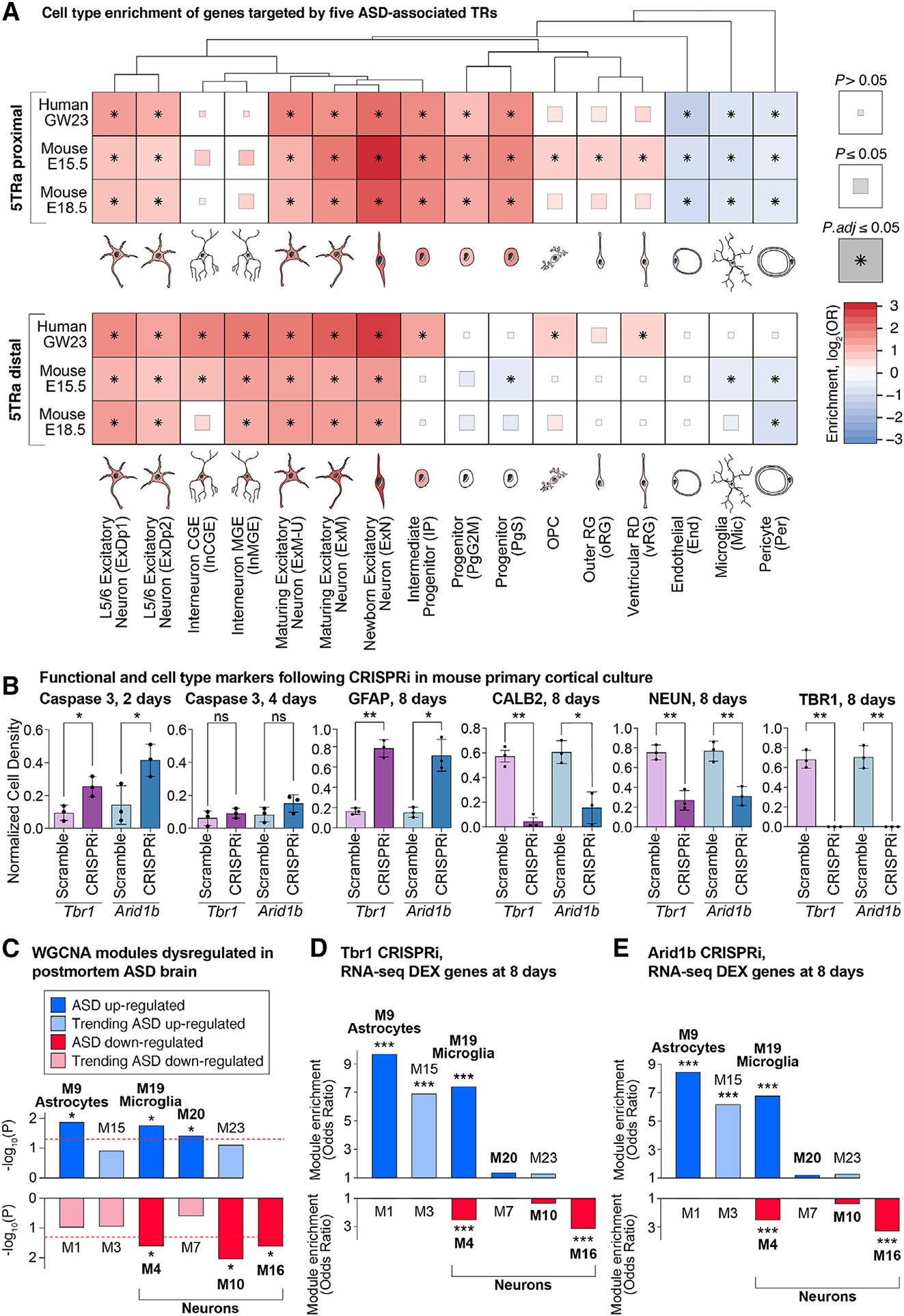
Cell-type enrichment and functional consequences (A) Cell-type clusters from the human fetal cortex^33^ were assessed for enrichment of genes targeted by all five ASD-associated TRs and ATAC-seq (5TRa) in human (GW23) and mouse (E15.5 and E18.5) fetal cortex. The degree of enrichment is indicated by color; the significance threshold is indicated by box size/asterisk. (B) Cortical cells from post-natal day 0 dCAS9-KRAB mice were infected with lentiviral sgRNAs to the promoters of *Arid1b*, *Tbr1*, or scrambled controls. Immunohistochemistry was used to compare the five markers. (C) Down- (red) and up-regulated (blue) modules of coexpressed genes were previously identified in the prefrontal cortex of individuals with ASD.^7^ (D) Differentially expressed genes, following CRISPRi to *Arid1b*, were identified from bulk RNA-seq of day 8 cultured cells. The enrichment of differentially expressed genes is shown in the modules from (C). (E) The analysis in (D) is repeated for CRISPRi to *Tbr1*. GW23, gestational week 23; E15.5/E18.5, embryonic days 15.5/18.5. Statistical analyses: (A) Fisher’s exact test; (B) two-tailed t test with Tukey correction was used for pairwise comparisons; (D and E) Fisher’s exact test; *p ≤ 0.05 and ***p ≤ 0.001. See also Figures S6 and S7.

ASD-associated genes are enriched within genes expressed by excitatory and inhibitory neurons from the human fetal cortex.^6,17,28^ Analysis of cells that express genes near 5TRa loci provides an orthogonal approach to identify the cell types involved in ASD. Therefore, we assessed cell-type enrichment in ~40,000 cells from the human fetal cortex at GW17–18.^33^ Across 5TRa loci in human and mouse fetal cortex, enrichment was seen for cortical progenitor cells and excitatory neurons at proximal loci and for excitatory and inhibitory neurons at distal loci (Figure 6A). Consistent results were observed for other fetal cortex transcriptomic datasets (Figure S7).

### Reduction of ARID1B and TBR1 expression in cultured neonatal mouse cortical cells reduces neuronal density and increases glial density, recapitulating human postmortem patterns of gene expression

The overlapping peaks of ASD-associated TRs suggests a convergent mechanism through which disruption of multiple genes can lead to similar neurodevelopmental phenotypes; this predicts similar functional consequences to changes of their expression. To test this prediction, we used lentivirus to deliver CRISPRi sgRNAs, designed to target proximal 5TRa loci in the promoter region of *Arid1b* or *Tbr1*, in primary cortical cultures collected from post-natal day (P) 0 dCAS9-KRAB mice. Compared to scrambled sgRNAs, CRISPRi reduced the relative expression of *Arid1b* to 75% by day 2 and to 25% by day 8; likewise, *Tbr1* was reduced to 50% by day 2 and to 5% by day 8 (Figure S6iii). CRISPRi to either *Arid1b* or *Tbr1* increased caspase-3 on day 2, but not day 4, suggesting a transient increase in apoptosis. By day 8, immunohistochemistry showed there was a reduction of neuronal marker expression (~3-fold reduction in NeuN^+^ cells and ~6-fold reduction of CALB^+^ cells), accompanied by an increase in astrocyte marker expression (~4.5-fold increase in GFAP^+^ cells; Figure 6B).

Bulk RNA sequencing (RNA-seq) analysis of the *Arid1b* and *Tbr1* CRISPRi-treated cells identified numerous differentially expressed genes (absolute fold change ≥ 2, adjusted *p* value [*p.adj*] ≤ 0.05, Table S7). To assess how these results related to patterns of gene coexpression module dysregulation observed in the postmortem cortex of individuals with ASD,^7^ we considered the enrichment of these modules for the differentially expressed genes (Figure 6C). Remarkably, both the *Arid1b* and *Tbr1* CRISPRi-treated cortical cells produced a pattern similar to that seen in ASD human brains. Neuronal modules that are down-regulated in the ASD brain (M4, M16) were enriched for down-regulated genes following CRISPRi (*p* ≤ 7 × 10^−9^, Figures 6D and 6E; Table S7). On the other hand, astrocyte and glial modules that are up-regulated in the ASD brain (M9, M19) were enriched for up-regulated genes following CRISPRi (*p* ≤ 9 × 10^−12^, Figures 6D and 6E; Table S7). While the direction of the effect was similar, the magnitude differed between the cellular assay and postmortem brain data.^8^ Cell-type deconvolution for six major cell types in postmortem human data suggests a 2.3% decrease in upper layer excitatory neurons in ASD compared to control (*p.adj* = 0.046, logistic regression with RIN, PMI, sex, and age as covariates, Figure S7), with other cell types differing by 3.4% or less, in contrast to the dramatic changes in cell-type composition in the CRISPRi primary cortical culture assay (Figure 6B).

## DISCUSSION

Over half the genes associated with ASD play a role in gene regulation, suggesting that transcriptional dysregulation is a major etiological factor in ASD.^2,34,35^ Here, we used ChIP-seq to identify regulatory targets of five of these ASD-associated TRs (ARID1B, BCL11A, FOXP1, TBR1, and TCF7L2) in the developing human and mouse cortex; we found that they converge on about 15,000 loci (6.5 Mbp) proximal to the TSSs of genes that are highly expressed in the developing brain, along with 5,000 distal loci (1.5 Mbp). This overlap is surprising, since four of the five genes have a distinct DNA-binding domain and known motif (Figure S2E), they belong to different transcription regulator classes, and they have variable expression patterns across tissues and cell types (Figure S7A). Despite this heterogeneity, we show that this overlap is greater than expected compared with ChIP-seq data from other TRs in heterogeneous tissues (Figures 2A and 2B) and is also observed at two developmental stages in the mouse cortex (Figures 2E–2I).

Our results provide a parsimonious explanation for how the disruption of multiple ASD-associated genes leads to a common diagnostic entity. Out of 102 ASD-associated genes,^2^ 101 have three or more of the ASD-associated TRs binding in open chromatin regions near their TSS (proximal ≥ 3TRa); 96 of these are targeted by all five ASD-associated TRs (proximal 5TRa, Figure 4). This provides a mechanism by which disruption of each gene can impart risk to ASD and by which this risk can converge to a shared phenotype across many genes. We predict that other ASD-associated genes with a role in transcriptional regulation will also follow this pattern.

While the overlap of ASD-associated TR binding sites provides a potential mechanism for convergent ASD risk, it presents a challenge for explaining specificity to ASD, since almost half of all protein-coding genes share the proximal 5TRa pattern. Considering epigenetic and transcriptomic data from the developing human cortex provides a potential solution. The ASD-associated TR binding sites overlap substantially with loci identified by ATAC-seq (open chromatin)^23^ and H3K27ac ChIP-seq but show minimal overlap with H3K27me3 ChIP-seq peaks (Figures 2C–2H); this suggests a predominant role in transcriptional activation. This conclusion is supported by the strong correlation between the number of ASD-associated TRs at proximal open chromatin sites and the level of gene expression in the developing human cortex.^6^ Analysis of single-cell transcriptomic data from the developing human cortex^17,33,36^ suggests that these observations arise from cells of the neuronal lineage (Figure 6A). From these data, we might predict that the predominant role of these ASD-associated TRs is to increase gene expression during the development of neuronal lineage cells. Since ASD genes are preferentially expressed in neuronal lineage cells^2,6,19^ and are among the most highly expressed genes in the cortex (Figure 4I), they may be especially vulnerable to perturbation of ASD-associated TRs. The binding of ASD-associated TRs at distal sites may further add to this vulnerability, as suggested by our analysis of VISTA element hs399 and its role in *BCL11A* expression (Figure 5).

Protein-protein interactions between the TRs could explain the shared genomic targets (Figure 2) and the reduced activity of the hs399 VISTA enhancer in the absence of TBR1 (Figure 5). Protein interaction data from the literature provide some support for this possibility, including interactions of BCL11A-TBR1,^37^ FOXP1-TBR1,^38^ FOXP1-BCL11A (via NR2F1/NR2F2),^39^ FOXP1-TCF7L2,^40^ and FOXP1-YY1 (Figure 3).^39^ Motif analysis results would also fit with this explanation. Four of the five ASD-associated TRs have previously been associated with a distinct DNA-binding motif based on DNA sequence enrichment within ChIP-seq peaks (Figure S2E). While we do observe enrichment of the BCL11A, FOXP1, TBR1, and TCF7L2 motifs (Figure S2E), the degree of enrichment is comparatively modest (Figure 3; Table S4). In contrast, other DNA-binding motifs, such as the motif of the ASD-associated genes *RFX3* and *TCF4*, are substantially enriched (Figure 3). These motif data also support the hypothesis that one of the core roles of these five TRs is to drive the expression of genes in neuronal lineage cells through binding to the promoter region. The observed motifs (Figure 3) and hs399 VISTA enhancer experiment (Figure 5) implicate the formation of promoter-enhancer loops in this role, based on the enrichment for the GFY-STAF-ZNF143 proximal motif and the CTCF/BORIS distal motif (Figure 3). These patterns of protein-protein interactions may reflect the role of these ASD-associated TR proteins in known chromatin regulatory complexes, including BAF/SWI/SNF (ARID1B, BCL11A, TCF7L2) and NuRD (BCL11A, FOXP1).^41–43^

If regulatory ASD-associated genes act through complexes at promoter and/or enhancer regions to increase gene expression in neuronal lineage cells, then we would predict that decreased expression of these target genes would confer ASD risk. This fits conceptually with exome-sequencing results, which identified ASD risk through loss-of-function variants in genes with high neuronal expression^2,6^ and whole-genome sequencing results, which implicate *de novo* variants in promoter regions conserved across species.^44^ Regulatory complexes also provide a mechanism by which multiple common variants of small effect size could lead to additive risk.^45,46^ Under this regulatory complex model, we would predict that heterozygous disruption of one of these ASD-associated TRs would lead to dysregulation of other ASD genes. This expectation is borne out in mice with heterozygous loss of function of *Foxp1*^47^; however, the interpretation is challenging due to experimental heterogeneity, including differences in brain region, developmental stage, and mutation and the varying sensitivity to detect differential gene expression based on baseline levels of gene expression.

As a complementary approach to assessing the functional impact of loss of function of ASD-associated TRs, we used CRISPRi to knock down *Arid1b* and *Tbr1* in primary cultures from P0 mouse neocortex. As predicted, we observed substantially decreased expression of numerous ASD-associated genes; corresponding increased expression was observed for genes in previously defined coexpression modules enriched for astrocytes and microglia marker genes (Figures 6D and 6E). These results mirror patterns observed from bulk and single-cell analyses of ASD cases and controls in the postmortem human brain.^7,9,48^ Concurrent analysis of cell-type markers suggests that these signals are driven by changes in cell-type proportion, specifically a relative reduction in neurons and an increase in astrocytes (Figure 6B). A corresponding transient increase in caspase-3 suggests increased apoptosis in the presence of the knockdown; thus, many of the observed transcriptional changes may reflect an alteration in the ratio of cell types.

Similar changes in cell-type proportion have been observed following the disruption of cortical transcription factors, including *TBR1*, in human organoid models,^49^ and an increase in the proportion of protoplasmic astrocytes was described in the postmortem cortex of ASD cases.^9^ Deconvolution of bulk transcriptomic data from the postmortem brain in ASD cases and controls shows similar patterns; however, the changes are modest and heterogeneous (Figure S7).^8^

Limitations to our analysis include the reliance on bulk cortical tissue to derive the ChIP-seq and ATAC-seq peaks, so we cannot directly differentiate between cell types. However, some cell-type information can be gleaned, since *BCL11A* and *TBR1* cortical expression appear to be specific to the neuronal lineage and *TBR1* is preferentially expressed in deep layer excitatory neurons,^20^ while *TCF7L2* is expressed in progenitor cells.^50^ Nonetheless, we cannot be sure that the observed overlaps in TR binding sites exist in individual cells vs. represent a pattern across multiple cell types. While the model of regulatory complexes bound to shared promoter and/or enhancer regions potentially explains convergence across TR ASD-associated genes, it is unclear how disruption of ASD-associated genes that are not thought to be TRs (e.g., *SCN2A, SYNGAP1, SLC6A1*) can lead to equivalent behavioral outcomes. However, the convergence of ASD gene function in regulating synaptic function, as seen for TBR1,^20,51^ is a parsimonious explanation. Furthermore, while this regulatory complex model predicts widespread down-regulation of highly brain-expressed genes, especially those associated with ASD, more data are required to verify this prediction. It is also unclear to what extent gene dysregulation or changes in the proportion of neuronal lineage cell types contribute to ASD symptomatology. Furthermore, it is unclear the extent to which a 75%–95% knockdown using CRISPRi in cultured mouse cortical neurons should reflect a 50% constitutive knockout in the developing human brain. These results raise a potential convergent downstream consequence of regulatory disruption, but substantial work is required to assess whether similar consequences occur in ASD or contribute to symptoms.

## Conclusions

Analysis of five ASD-associated transcription regulators leads to a model in which their encoded proteins act as components of molecular mechanisms titrated to control gene expression in developing neuronal lineage cells. Like a clock mechanism, many components are essential, and the failure of any individual component can impact overall function. Under this model, disruption of any of multiple ASD-associated TR genes leads to a common neurodevelopmental outcome through shared genomic targets, while specificity to ASD and developmental delay is due to a combination of haploinsufficiency and high neuronal expression of ASD-associated target genes during neurodevelopment, making them the most vulnerable to small perturbations in expression.

## STAR★METHODS

### RESOURCE AVAILABILITY

#### Lead contact

Further information and requests for resources and reagents should be directed to and will be fulfilled by the Lead Contacts, Dr. Stephan Sander (stephan.sanders@paediatrics.ox.ac.uk) and Dr. John L. Rubenstein (john.rubenstein@ucsf.edu).

#### Materials availability

All unique/stable reagents generated in this study are available from the lead contacts without restriction.

#### Data and code availability

- The data used in this publication have been deposited in NCBI’s Gene Expression Omnibus (GEO) under accession number GSE248876 (https://www.ncbi.nlm.nih.gov/geo/query/acc.cgi?acc=GSE248876).
- All original code has been deposited at https://github.com/sanderslab/five_tr_chip and is publicly available as of the date of publication.
- Any additional information required to reanalyze the data reported in this paper is available from the lead contact upon request.

### EXPERIMENTAL MODEL AND STUDY PARTICIPANT DETAILS

#### Animals

All procedures and animal care were approved and performed in accordance with the University of California San Francisco Laboratory Animal Research Center (LARC) guidelines. All wildtype strains were maintained on a CD1 background. Animals were housed in a vivarium with a 12hr light, 12hr dark cycle. Postnatally, experimental animals were kept with their littermates. For timed pregnancies, noon on the day of the vaginal plug was counted as embryonic day 0.5.

#### Transgenic animal models

dCAS9-KRAB mouse was a gift from McManus lab (UCSF) on a CD1 background. The dCas9-KRAB mice were generated in the FVB background with the TARGATTTM site-specific knock-in technology^53^ by introducing a construct expressing containing CAG promoter, puromycin resistance, mCherry and the dead Cas9 (dCas9) protein fused to the KRAB (Kruppel Associated Box) domain into€ the *Hipp11* locus. hs399-CT2IG enhancer transgenic mouse was generated at the Gladstone Transgenic Gene Targeting Core.

### METHOD DETAILS

#### Transcription regulator chromatin immunoprecipitation and sequencing (TR ChIP-Seq)

Transcription regulator ChIP (TR ChIP) was performed using antibodies (specified below) against ARID1B, BCL11A, FOXP1, TBR1, and TCF7L2. Wildtype mouse cortices were dissected from E15.5 and E18.5 brains in ice-cold HBSS.

De-identified tissue samples were obtained with patient consent in strict observance of the legal and institutional ethical regulations. Protocols were approved by the Human Gamete, Embryo, and Stem Cell Research Committee, the institutional review board at the University of California San Francisco (UCSF). Fresh fetal brain samples were obtained from elective terminations, with no karyotype abnormalities or genetic conditions reported, and transported in freshly made Cerebral Spinal Fluid on ice (CSF). Samples were collected from gestational week 23 (GW23) prefrontal cortex (PFC). All dissections and ChIP-seq experiments were performed within 2 h of tissue acquisition.

Human and mouse cortical samples were dissociated by pipetting in ice-cold HBSS. Dissociated cells were fixed in 1% formaldehyde for 10 min at RT. The fixed cells were neutralized with 1 mL 2.5M glycine and washed 3X in 1X PBS on ice. Fixed cells were lysed in a hypotonic buffer (50 mM Tris-HCl pH = 7.5, 0.5% NP-40, 0.25% Sodium Deoxycholate, 0.1% SDS, 150 mM NaCl). Nuclei was extracted by centrifugation at 13,500 rpm for 10 min at 4°C and sheared into 200 – 1,000 bp fragments using a Covaris S2 (14 cycles of duty cycle = 5%, intensity = 3 and cycles per burst = 200).

Immunoprecipitation (IP) reactions of two biological replicates on mouse cortex at E15.5 and E18.5 and human PFC at GW23 were performed by diluting the sheared chromatin 1:10 in ChIP dilution buffer (16.7 mM Tris-HCl pH = 8.0, 1.2 mM EDTA, 167 mM NaCl, 0.01% SDS, 1.1% Triton X-100) in 3 mL final volume. 100 μL was removed as “input”. 5 μg of primary antibody against ARID1B (Santa Cruz Biotech, sc32762 X), BCL11A (Abcam, ab19487), FOXP1 (Santa Cruz Biotech, sc-376650 X), TBR1 (Santa Cruz Biotech, sc48816 X) and TCF7L2 (Santa Cruz Biotech, sc166699 X) were added to each IP. 20X molar excess blocking peptide (FOXP1) and IgG (ARID1B, BCL11A, TCF7L2) and TBR1 constitutive null were used as negative control for each given ChIP experiments.

Antibody specificity has been examined for all the target proteins. The antibody specificity against TBR1,^20^ BCL11A^54^ and ARID1B^55^ were previously demonstrated. We assessed anti-FOXP1 antibody specificity using a blocking peptide designed against the antibody epitope (Santa Cruz Biotech, sc-376650 P). TCF7L2 antibody specificity was examined through IHC analysis the TCF7L2 staining in the Tcf7l2^f/f^:1538CRE-ERtdTomato^f/+^ conditional mutant compared to the heterozygous control (Tcf7l2^f/+^1538CRE-ERtdTomato^f/+^) at E12.5. Using this paradigm, we observed TCF7L2 signal reduced in pre-optic area (POA) in the conditional null mutant compared to the heterozygous controls, a region where CRE-expressing lineage cells are present.

Protein/antibody complexes were collected using Dynabeads (20 μL protein A + 20 μL protein G). Beads were washed once in each of Wash buffers (low salt buffer: 0.1% SDS, 1% Triton X-100, 2 mM EDTA, 20 mM Tris-HCl pH = 8.0, 150 mM NaCl; High Salt buffer: 0.1% SDS, 1% Triton X-100, 2 mM EDTA, 20 mM Tris-HCl pH = 8.0, 500 mM NaCl; LiCl buffer: 0.25 M LiCl, 1% IGEPAL CA630, 1% deoxycholic acid, 1 mM EDTA, 10 mM Tris-HCl pH = 8.0 and lastly 1X TE pH = 7.5. ChIP DNA was eluted twice in elution buffer (10 mM Tris-HCl pH = 8.0, 1 mM EDTA, 100 mM NaHCO_3_, 1 mM SDS) at 65°C for 15 min each with shaking. Eluted ChIP DNA was reverse cross-linked in 8 μL 5M NaCl, 4 μL 1M Tris-HCl pH = 6.5, 4 μL 0.5M EDTA overnight at 65°C. ChIP DNA was treated with 4 μL 10 mg/mL RNase A at 37°C for 15 min and then 1 μL 10 mg/mL proteinase K to each sample and incubated at 55°C for 1 h.

ChIP DNA was purified using ChIP DNA Clean and Concentrator kit (Zymo Research, D5205). ChIP-seq libraries were generated using Ovation Ultralow System V2 Multiplex System (NuGEN) following manufacturer’s protocol, using 12 PCR cycles. The resulting libraries were size selected 180–350 bp using BluePippin (Sage Science) and sequenced at the Center for Advanced Technology at UCSF (Illumina HiSeq 4000; http://cat.ucsf.edu/) using a single read 50-bp strategy.

#### Histone chromatin immunoprecipitation and sequencing (histone ChIP-Seq)

Human and mouse samples were acquired as for TR ChIP-seq above. All dissections and downstream experiments were performed within 2 h of tissue acquisition. From each dissection, nuclei were isolated by manually douncing the tissue twenty times in 1 mL Buffer I (300 mM sucrose, 60 mM KCl, 15 mM NaCl, 15 mM Tris-HCl pH = 7.5, 5 mM MgCl_2_, 0.1 mM EGTA, 1 mM DTT, 1.1 mM PMSF, 50 mM Sodium Butyrate, EDTA-free Protease inhibitors) on ice using a loose pestle douncer, and then lysed on ice for 10 min after adding 1 mL Buffer II (300 mM sucrose, 60 mM KCl, 15 mM NaCl, 15 mM Tris-HCl pH = 7.5, 5 mM MgCl_2_, 0.1 mM EGTA, 0.1% NP-40, 1 mM DTT, 1.1 mM PMSF, 50 mM Sodium Butyrate, EDTA-free Protease inhibitors).

During the incubation, nuclei were counted using trypan blue and 500,000 nuclei were spun down at 7,000rpm for 10 min at 4°C. Nuclei were resuspended in 250 μL MNase buffer (320 mM sucrose, 50 mM Tris-HCl pH = 7.5, 4 mM MgCl_2_, 1 mM CaCl_2_, 1.1 mM PMSF, 50 mM Sodium Butyrate) and incubated in a 37°C water bath with 2 μL MNase enzyme (NEB) for 8 min. MNase digestion was stopped by adding 10 μL 0.5M EDTA, and chromatin was spun down for 10 min at 10,000 rpm 4°C. Soluble fraction S1 supernatant was saved at 4°C overnight, and S2 fraction was dialyzed overnight in 250uL dialysis buffer at 4C (1 mM Tris-HCl pH = 7.5, 0.2 mM EDTA, 0.1 mM PMSF, 50 mM Sodium Butyrate, 1X Protease Inhibitors).

S1 and S2 fractions were combined, 50 μL was saved as input, and immunoprecipitation assay was set up in 50 mM Tris-HCl pH = 7.5, 10 mM EDTA, 125 mM NaCl, 0.1% Tween 20. 250 mM Sodium Butyrate was supplemented for H3K27ac ChIPs. The following antibodies were used for ChIP: H3K27ac (Millipore, cma309), H3K4me1 (Abcam, ab8895), H3K27me3 (Millipore, 07–449), H3K4me3 (Abcam, ab185637). 1 mL of antibody was added to 1 mL chromatin in ChIP dilution buffer (16.7 mM Tris-HCl pH = 8.0, 1.2 mM EDTA, 167 mM NaCl, 0.01% SDS, 1.1% Triton X-100) and incubated overnight with chromatin at 4C rotating. Protein A and Protein G beads (10 μL each) were blocked overnight in 700 μL ChIP buffer, 20 μL yeast tRNA (20 mg/mL), and 300 μL BSA (10 mg/mL). Beads were washed three times on ice in Wash buffer I (50 mM Tris-HCl pH = 7.5, 10 mM EDTA, 125 mM NaCl, 0.1% Tween 20, supplemented with 1X protease inhibitors and 5 mM sodium butyrate) and three times in Wash buffer II (50 mM Tris-HCl pH = 7.5, 10 mM EDTA, 175 mM NaCl, 0.1% NP-40, supplemented with 1X protease inhibitors and 5 mM sodium butyrate). Lastly, beads were washed once in 1X TE buffer. ChIP DNA was eluted twice in 100 μL elution buffer (10 mM Tris-HCl pH = 8.0, 1 mM EDTA, 100 mM NaHCO_3_, 1 mM SDS) at 37°C for 15 min each with shaking.

IP reactions were incubated at 65°C for 30 min and purified using ChIP DNA Clean and Concentrator kit (Zymo Research, D5205). ChIP-seq libraries were generated using Ovation Ultralow System V2 (NuGEN) following manufacturer’s protocol. The resulting libraries were size selected (180–350 bp) and sequenced at the Center for Advanced Technology at UCSF (Illumina HiSeq 4000; http://cat.ucsf.edu/) using a single read 50-bp strategy.

#### ChIP-seq Computational analysis

##### Peak Calling

Human samples were aligned to the main chromosomal contigs of the GRCh38 genome. Mouse samples were aligned to the main chromosomal contigs GRCm38 (mm10) genome. Both alignments were performed by BWA (v0.7.15) *bwa mem ref_genome.fa sample.fastq.gz*. Resulting SAM files were converted to BAM files with a MAPQ filter of 30 and sorted using *samtools (v1.10). samtools view -q 30 -Shu -o sample.unsorted.bam sample.unsorted.sam*, and *samtools sort -o sample.bam sample.unsorted.bam*.

For all phenotypes, significant peaks were identified against matched input controls or WT background using mac2 (v2.2.7.1). Narrow peak calling was used with a q-value cut-off of 0.01. Model-based peak calling and local significance testing were disabled. A fixed fragment extension length of 200bps was used. *macs2 callpeak -t sample.bam -c input.sample.bam -f BAM -g mm/hs -no-lambda -nomodel -ext 200 -bdg -q 0.01*. The data used in this publication have been deposited in NCBI’s Gene Expression Omnibus (GEO) under accession number GSE248876 (https://www.ncbi.nlm.nih.gov/geo/query/acc.cgi?acc=GSE248876).

For mouse samples, biological replicates were kept separate when first identifying peaks. IDR analysis was used to confirm the quality of peaks between biological replicates. Aligned reads of the replicates were then merged and new peaks were called. The peaks derived from merged biological replicates were used for downstream analysis.

##### Coverage heatmaps

Coverage heatmaps for transcription factor ChIP-seq samples (Figure 1) were generated using deepTools (v3.5.1). Biological replicates were kept separate in these heatmaps to better display coverage. The regions shown were pooled from peaks from every transcription factor of the designated age. The viewing reference point was set to center and a viewing range of 1kb was used. *computeMatrix reference-point -referencePoint center -S [sample1.bigwig, sample2.bigwig, … sampleN.bigwig] -R E15_peaks.bed -o E15_matrix.txt -a 1000 -b 1000. Followed by, plotHeatmap -m E15_matirx.txt -o E15_heatmap.pdf -missingDataColor white*.

##### Peak annotation

Called peaks from the Chip-seq datasets were annotated to all transcripts on GENCODE version 31 (human; hg38) and GENCODE version 23 (mouse; mm10). Proximal peaks were defined as being 2,000bp upstream of the TSS. Where multiple TSS were present for a given gene, we used the union of all proximal regions. BedTools intersect was used to identify overlap between peaks and gene promoters^56^ with any overlap between the ChIP-seq peak and promoter region defining the peak as “proximal”. Chip-seq peaks that did not overlap with promoter regions were defined as “distal” and the nearest TSS, identified by BedTools closest, was used to define the gene associated with distal peaks (Figure 1).

#### *In vitro* CRISPRi assay in mouse primary cortical cultures

##### Guide design

To generate lentiviral guide constructs against Transcriptional Start Site (TSS) of *Arid1b* and *Tbr1* using 2 kb upstream of TSS for each given gene. In addition to TSS-sgRNA, lentiviral guide constructs were also designed against putative regulatory elements (pREs), each of which are bound *in vivo* by all hcASD-TRs, forming “hubs”. The DNA sequences were inputted into CRISPOR tool^57^ from Zhang lab at Massachusetts Institute of Technology (https://zlab.bio/guide-design-resources). To facilitate the cloning process, TTGG and CAAA were added to 5′ and 3′-ends of forward and reverse guides, respectively. Scrambled guides were designed using GenScript online browser tool from *Arid1b* and *Tbr1* TSS-sgRNAs (https://www.genscript.com/tools/create-scrambled-sequence). The complete list of guides and the corresponding genomic regions are shown below.

**Table:** 

Gene	Location	Forward sequence	Reverse sequence

*Arid1b*-TSS	chr17:4994464-4995567	TTGG**CCATGTTCAGGTCGTGACGG**	AAAC**CCGTCACGACCTGAACATGG**
*Tbr1*-TSS	chr2:61803569-61804574	TTGG**TATACAAAGCGCGAGCCGG**	AAAC**CCGGCTCGCGCTTTGTATA**
*Bcl11a*-TSS	chr11:24049187-24049941	TTGG**TGGGAGAGCTCCATATGGCA**	AAAC**TGCCATATGGAGCTCTCCCA**
scrambled-*Arid1b*-TSS	–	TTGG**TTGCGGACTTGGTACGACC**	AAAC**GGTCGTACCAAGTCCGCAAC**
scrambled-*Tbr1*-TSS	–	TTGG**AGCGAGCGTAGAGCGCAATC**	AAAC**GATTGCGCTCTACGCTCGCT**

##### Generating sgRNA lentivirus

Guide RNA oligonucleotides were annealed in 1X annealing buffer (100 mM Tris pH7.5, 1 M NaCl, 10 mM EDTA) by heating for 5 min at 95°C, then cooling down gradually to 25°C, 5 °C/min. *U6-stuffer-longTracer-GFP* lentivirus vector was digested with AarI enzyme overnight and gel purified. The annealed guides were cloned into the digested *U6-stuffer-longTracer-GFP* lentivirus vector overnight at 16°C. Ligated guides were cloned into *Stbl3* cells (Thermofisher) and verified by sequencing at ElimBio using Elim Primer# 258124.

Upon sequencing validation, the gRNA lentivirus was generated in HEK293T cells by transfecting 3′10 cm dishes with 3 μg sgRNA plasmid, 1.5 μg psPAX2 packaging vector (Addgene), 1.5 μg pmD2G envelope vector (Addgene), 850 μL jetPRIME buffer and 18 μL jetPRIME. Three days post-transfection, the sgRNA expressing lentivirus was purified as described previously.^58^ Empty *U6-stuffer-longTracer-GFP* lentivirus vector was used to generate mock control lentivirus.

##### Primary cell culture and In vitro CRISPRi assay

Cortex was dissected from P0 dCAS9-KRAB pups and dissociated using papain dissociation kit following manufacturer’s protocol (Worthington). A total of 400,000 cells were seeded into 24-well tissue culture dishes containing fresh N5 medium (500 μL N2 supplement, 121 μL BPE (bovine pituitary extract), 10 μL 100 ng/uL FGF, 10 μL 100 ng/uL EGF, 5 mL FBS, 0.5 mL Pen/Strep in 50 mL DMEM) that were pre-coated with poly-L-lysine (10 mg/mL, Sigma) and then laminin (5 mg/mL, Sigma). Polybrene (Thermofisher) was added to each tube to facilitate transduction at a final concentration of 8 μg/mL. Concomitantly, the P0 dCAS9-KRAB cells were transduced by adding 20 μL concentrated virus at the time of seeding. The cultures were grown for 16 days *in vitro* and N5 media was replaced every 48 h. This experiment was repeated twice (*n* = 2).

#### RNA extraction and cDNA synthesis

Total RNA was extracted from the primary cortical cultures at 2, 4, 8, 12 and 16 days-post-transduction (DPT) using RNeasy Plus Micro Kit (QIAGEN, Cat# 74034) following the manufacturer’s protocol. First strand cDNA was synthesized from 0.5 μg of total RNA using Superscript reverse transcriptase III following manufacturer’s protocol (Thermofisher).

#### Quantitative real-time PCR (qPCR)

Quantitative real-time PCR (qPCR) was performed to measure RNA levels using SYBR Green (Bio-Rad) and 7900HT Fast Real-Time PCR System. Gene-specific primers for exon #1 of *Arid1b, Bcl11a, Tbr1, Drd1, Hbb, eif1α* as well as *Gapdh* housekeeping genes (HKG) were designed using the Primer 3 program. The expression levels of the genes in mock control and each TSS-CRISPRi RNA were normalized to the expression levels of the HKGs. Subsequently, the gene expression levels in TSS-CRISPRi RNAs were measured relative to the mock control using DDCT method as previously described (^59,60^) and averaged across 3 experimental replicates for each biological set (*n* = 2 biological replicates).

**Table:** 

Gene	Forward Primer	Reverse Primer

*Arid1b*	GCGCAACAAAGGAGTCACC	CCCATCCCATACAACTGAGG
*Bcl11a*	CACAAACGGAAACAATGCAA	CACAGGATTGGATGCCTTTT
*Tbr1*	CCCAATCACTGGAGGTTTCA	GAGATTTCTTGCCGCATCCA
*Drd1*	GAAGATGCCGAGGATGACAAC	GGCTACGGGGATGTAAAAGC
*Hbb*	GCTGGTTGTCTACCCTTGGA	GGCCTTCACTTTGGCATTAC
*Eif1a*	AAGCTCTTCCTGGGGACAAT	ATGCTATGTGGGCTGTGTGA
*Gapdh*	CCGTAGACAAAATGGTGAAGG	CAATCTCCACTTTGCCACTGC

#### Generation of hs399-CT2IG enhancer transgenic mouse

Enhancer hs399 was amplified from human genomic DNA and subcloned into Hsp68-CreERT2-IRES-GFP.^61^ Stable transgenic mice were generated by pronuclear injection at the Gladstone Transgenic Gene Targeting Core using the FVB strain. Founders were screened by PCR.^32^

#### RNA-seq on TSS-CRISPRi cells

Transcriptome profiling was conducted by using RNA-seq on *in vitro* dCAS9-KRAB (CRISPRi) cells 8 days-post-transduction by lentivirus encoding sgRNA guides against TSS of *Arid1b* and *Tbr1*. Approximately 300,000 cells were collected from each sample and immediately proceeded with RNA extraction using RNeasy Plus Micro Kit (QIAGEN) following manufacturer’s protocol. RNA quality was assessed using Agilent RNA 6000 Nano Kit (Agilent Technologies) and ran on Bioanalyzer 2100 (Agilent Technologies). Samples that had RIN scores of 8.5–10 were used to generate libraries. Library preparation and amplification was performed by TruSeq Stranded Total RNA Library Prep Kit with Ribo-Zero Gold Set A (Illumina, Cat# RS-122–2001). The amplification of adapter-ligated fragments was carried out for 12 cycles during which individual index sequences were added to each distinct sample. Library concentration was assessed with Qubit (Thermofisher, Cat# Q33231) and library fragment size distribution was assessed on the Agilent Bioanalyzer 2100 (Agilent Technologies) and Agilent High Sensitivity DNA Kit (Agilent Technologies) following manufacturer’s protocol. Pooled, indexed RNA-seq libraries were sequenced on HiSeq 4000 at Center for Advanced Technology (Illumina HiSeq 4000; http://cat.ucsf.edu/) to produce 150 bp paired-end reads.

Read count and transcript per million reads mapped (TPM) were determined using Salmon software version 1.3.0. A reference genome index for Salmon was created according to developer’s instructions for the mouse reference transcriptome from the GENCODE v23. Reads mapping and quantitation was simultaneously performed to individual transcripts. Gene-level counts were collated and normalized using the tximport and DESeq2 R Bioconductor packages (^62,63^). We excluded the genes with zero count and selected the genes used in the enrichment analysis. Differential gene expression (DGE) analyses were performed on two replicates of TR TSS-CRISPRi against five control libraries, with following: ~ replicate + condition. With random selections of control libraries, we did confirm our DGE consistently generate fold changes and statistics regardless of control sample batches. For gene set enrichment analysis, we used differentially expressed (DEX) genes of TSS-CRISPRi as those having adjusted *p*-value ≤0.05 and fold-change greater or less than 2, compared to control libraries. We collected a range of gene lists for ASD neurobiology and cortical development (^7,33,36^). DEX genes were converted to human ortholog genes using the HGNC annotation. Gene enrichment tests were assessed with a gene list using Fisher’s exact test with Bonferroni correction.

### QUANTIFICATION AND STATISTICAL ANALYSIS

#### ENCODE ChIP-seq overlap analysis

Where replicate samples were present the replicate with the highest number of total peaks was selected (ENCODE ENCFF002EXB, ENCFF132PDR, ENCFF765EAP, ENCFF039EYW, ENCFF366KUG, ENCFF215GBK, ENCFF934JOM, ENCFF264HRE, ENCFF643ZXX, ENCFF390HDY, ENCFF551VXN, ENCFF996EBR, ENCFF634YGY, ENCFF751TBY). FASTQ files were processed to generate peaks using the methods described above (‘Peak Calling’). Similar numbers of total proximal and distal peaks were present in the ENCODE liver and ASD-associated TR cortex samples (21,295 proximal peaks in liver vs. 23,668 in cortex, *p* = 0.61 Wilcoxon; 52,899 distal peaks in liver vs. 45,429 in cortex, *p* = 0.61 Wilcoxon). Peaks lists were sorted by *p*-value, followed by genomic location (if duplicate *p*-values were present) and the top 10,000 proximal peaks (selected due to 10,746 proximal peaks in ZBTB33) and 8,000 distal peaks (selected due to 8,002 distal peaks in FOXP1) were selected to exclude peak count as a variable. Bedtools intersect^56^ was used to identify the number of peaks at the same genomic location (any overlap was counted due to the large search space and to minimize the impact of peak size), expressed as a percentage of the peaks used (10,000 or 8,000). For peaks that did intersect, the correlation between the *p*-value and genomic location ranked lists was used to estimate Spearman’s rho.^24^ Similar patterns of overlap were observed when reducing the number of peaks used to 4,000 for both proximal and distal loci (Figure 2).

#### Overlap between epigenetic datasets

The union of ChIP-seq loci were split into proximal and distal loci, as described for the comparison to ENCODE. Overlap between these loci and epigenetic datasets (ATAC, H3K27ac, H3K27me3) was assessed with the Bedtools intersect function.^56^ Percentage overlap was used to calculate the number of overlapping nucleotides (Figure 2). Intersection was also used to assess overlap between peaks binned by proximal/distal status and any degree of overlap with ATAC-seq peaks (Figure 2). Peaks were considered overlapping between the five genes if there was any degree of nucleotide overlap. The fraction of total peaks overlapping between the five genes was used to determine color gradient (Figure 2).

#### Overlap between Phastcons loci

Phastcons hg38 100-way conservation scores were downloaded from UCSC genome browser and compared to ChIP-seq loci using the Bedtools intersect function.^56^ The maximum Phastcons score within a loci was used to assess conservation across species in a violin plot generated using the *Seaborn* package in Python (Figure 2). Cross species conservation of ChIP-seq peaks were estimated using UCSC Liftover with 1% minimal overlap.

#### Motif analysis

Enrichment for the primary motifs was assessed with Homer (v4.10.3) with default settings: findMotifs.pl *ChIP.narrowPeak.fa fasta consensus_motif -mknown select_motifs.txt*. No consensus motif was available for ARID1B. To assess genomic expectation, two definitions were used: genome-wide (window size of 200 and the repeated-masked sequence options) and loci targeted by ATAC-seq only and/or one ChIP-seq target only (nullTF). Motif enrichment was assessed using Homer (v4.10.3): *findMotifsGenome.pl 5TFa_NarrowPeaks.fa hg38/mm10 motif.out -size 200 -mask -gc or findMotifs.pl 5TFa_NarrowPeaks.fa fasta name -fasta nullTF_NarrowPeaks.fa*. The output table knownResults.txt was used to generate the motif enrichment plot (Figure 3).

#### Brain expression

Estimates of brain expression were obtained used transcripts per million (TPM) from developing human cortex data.^6^ For each protein-coding gene expressed in the brain, the overlap with proximal (+/− distal) ChIP-seq and ATAC-seq peaks was assessed. Log_2_(TPM+1) values were plotted as a violin plot generated using the *Seaborn* package in Python (Figure 4). Differences between proximal 5TRa and proximal non-5TRa were assessed using logistic regression.

#### Vista enhancer validation of distal ≥3TRa peaks

To determine whether ≥3TRa peaks are functional enhancers within the developing brain, we calculated the odds ratio of a locus overlapping a validated VISTA enhancer based on the presence or absence of a distal ≥3TRa peak. A bedtools intersect was performed on the list of 1,942 human VISTA enhancer regions and 13,875 distal ≥3TRa peaks. The VISTA enhancer atlas records the activity of each region across over 20 different tissues. The odds of being a positive VISTA enhancer in a given tissue based on the presence of a distal ≥3TRa peak was assessed via a two-sided Fisher’s exact test. Tissues with fewer than 50 positive VISTA enhancers were excluded from the analysis due to low sample size. The presence of a distal ≥3TRa peak significantly increased the odds of being a validated forebrain (OR = 2.17, FDR = 5.5 ×10^−5^, Table S6) or neural tube (OR = 2.27, FDR = 5.0 ×10^−4^, Table S6) enhancer (Figure S6A). A subset of VISTA forebrain enhancer sequences were retested *in vivo* and annotated by an expert developmental neuroanatomist for specific expression in the pallium, subpallium, or both and the analysis was repeated focusing on this subset alone (Figure S6B).

#### Identification of distal TR interactions with ASD genes via ABC score

The Activity-by-Contact (ABC) model identifies enhancer-gene relationships based on chromatin state and conformation and is more effective than distance-based approaches.^29^ Gestational week 18 (GW18) bulk ATAC-seq and H3K27ac ChIP-seq data from human fetal prefrontal cortex^23^ were aligned to hg19 using the standard Encode Consortium ATAC-seq and ChIP-seq pipelines respectively with default settings and pseudo replicate generation turned off (https://github.com/ENCODE-DCC). HiC contacts with 10kb resolution from human GW17–18 fronto-parietal cortex were obtained from http://resource.psychencode.org/Datasets/Pipeline/HiC_matrices/PIP-01_DLPFC.10kb.txt.tar.gz,^30^ in an hdf5 format separated by chromosome. Hdf5 files were filtered for contacts with a score >0 and converted into a bedpe format. Trimmed, sorted, duplicate and chrM removed ATAC-seq, sorted, duplicate removed ChIP-seq bam files, and HiC bedpe files from GW17–18 cortex were provided as input for calculating ABC scores.

According to the ABC score pipeline, (https://github.com/broadinstitute/ABC-Enhancer-Gene-Prediction) ATAC-seq and H3K27ac ChIP-seq bam files were provided as input to the MakeCandidateRegions.py script with the flags –peakExtendFromSummit 250 –nStrongestPeaks 150000. Candidate enhancer regions identified were then provided to the run.neighborhoods.py script in addition to hg19 transcript bounds merged by overlapping 2000bp promoters (Gencode v38lift37, basic). Finally, predict.py was used to identify final candidate enhancers using HiC data with the flags –hic_type bedpe –hic_resolution 10000 –scale_hic_using_powerlaw –threshold .02 –make_all_putative. All other settings for the ABC score pipeline remained constant.

To compare nearest neighbor vs. ABC approaches for identifying enhancer-gene pairs, we identified the nearest protein coding gene (Gencode v31, all) to each of the 13,875 identified distal ≥3TRa peaks (ARID1B, BCL11A, TBR1, and ATAC-seq) using bedtools closest with the options “-d -t first”. Candidate enhancer-gene pairs from ABC score were converted to hg38 using UCSC liftOver and liftOverBedpe (https://github.com/dphansti/liftOverBedpe). Overlaps between ≥3TRa peaks and candidate enhancer-gene pairs were identified using bedtools intersect. In total, 269 distal ≥3TRa peaks were nearest neighbors to 54 unique ASD genes, and 285 peaks had ABC contacts to 77 ASD genes.

#### CRISPRi analyses

Individual data points are shown as well as mean ± SEM. Statistical analyses were performed using GraphPad Prism 7.0 software. Statistical significance was accepted at the level *p* < 0.05. We used student’s t-test to compare pairs of groups if data were normally distributed (verified using Lillie test). If more than two groups were compared, we used one-way ANOVA with post-hoc tests between groups corrected for multiple comparisons (Holm-Sidak or Tukey). For the CRISPRi experiments reported in this paper (Figure 5C), *n* = 2 represents two biological replicates for each of the reported genes. The specific n for each experiment as well as the post-hoc test, exact F and corrected *p* values can be found in the Results section.

## Supplementary Material

1

2

3

4

5

6

7

8

9

## Figures and Tables

**KEY RESOURCES TABLE:** 

REAGENT or RESOURCE	SOURCE	IDENTIFIER

Antibodies		

Anti-TBR1 antibody	Santa Cruz Biotech	Cat# sc-48816 X
Anti-ARID1B antibody	Santa Cruz Biotech	Cat# sc-32762 X
Anti-FOXP1 antibody	Santa Cruz Biotech	Cat# sc-376650 X
Anti-TCF7L2 antibody	Santa Cruz Biotech	Cat# sc-166699 X
Anti-BCL11A antibody	Abcam	Cat# ab19487 RRID: AB_444947
FOXP1 Blocking Peptide	Santa Cruz Biotech	Cat# sc-376650 P
Goat anti-Mouse Alexa Fluor 647	Thermofisher Scientific	Cat# A32728 RRID: AB_2633277

Bacterial and Virus Strains		

Stbl3	Thermofisher	Cat# C737303

Chemicals, Peptides, and Recombinant Proteins		

Sucrose	Sigma Aldrich	Cat# S5016
Sodium bicarbonate (NaHCO_3_)	Sigma Aldrich	Cat# S6014
Glucose	Sigma Aldrich	Cat# G5767
Magnesium sulfate (MgSO_4_)	Sigma Aldrich	Cat# 230391

Critical Commercial Assays		

Bioanalyzer High Sensitivity DNA Kit	Agilent	Cat# 5067-4626
Bioanalyzer RNA 6000 Nano Kit	Agilent	Cat# 5067-1511
RNeasy Plus Micro Kit	QIAGEN	Cat# 74034
ChIP DNA Clean and Concentrator kit	Zymo Research	Cat# D5205
Ovation Ultralow System V2 Multiplex System	NuGEN	Cat# 0344
BluePippin	Sage Science	Cat# BDF2010

Deposited Data		

ChIP-seq Raw and Analyzed Data	NCBI’s GEO	Accession number GSE248876

Experimental Models: Cell Lines		

Mouse primary cortical culture	This paper	N/A
HEK293 cells	Thermofisher Scientific	Cat# R79007

Oligonucleotides		

*Arid1b* sgRNA forward sequence: TTGG**CCATGTTCAGGTCGTGACGG**	This paper	N/A
*Arid1b* sgRNA reverse sequence: AAAC**CCGTCACGACCTGAACATGG**	This paper	N/A
*Arid1b* scrambled sgRNA forward sequence: TTGG**TTGCGGACTTGGTACGACC**	This paper	N/A
*Arid1b* scrambled sgRNA reverse sequence: AAAC**GGTCGTACCAAGTCCGCAAC**	This paper	N/A
*Tbr1* sgRNA forward sequence: TTGG**TATACAAAGCGCGAGCCGG**	This paper	N/A
*Tbr1* sgRNA reverse sequence: AAAC**CCGGCTCGCGCTTTGTATA**	This paper	N/A
Scrambled sgRNA forward sequence: TTGG**AGCGAGCGTAGAGCGCAATC**	This paper	N/A
Scrambled sgRNA reverse sequence: AAAC**GATTGCGCTCTACGCTCGCT**	This paper	N/A
*Bcl11a* sgRNA forward sequence: TTGG**TGGGAGAGCTCCATATGGCA**	This paper	N/A
*Bcl11a* sgRNA reverse sequence: AAAC**TGCCATATGGAGCTCTCCCA**	This paper	N/A
*Arid1b* qPCR forward primer: GCGCAACAAAGGAGTCACC	This paper	N/A
*Arid1b* qPCR reverse primer: CCCATCCCATACAACTGAGG	This paper	N/A
*Bcl11a* qPCR forward primer: CACAAACGGAAACAATGCAA	This paper	N/A
*Bcl11a* qPCR reverse primer: CACAGGATTGGATGCCTTTT	This paper	N/A
*Tbr1* qPCR forward primer: CCCAATCACTGGAGGTTTCA	This paper	N/A
*Tbr1* qPCR forward primer: GAGATTTCTTGCCGCATCCA	This paper	N/A
*Drd1* qPCR forward primer: GAAGATGCCGAGGATGACAAC	This paper	N/A
*Drd1* qPCR reverse primer: GGCTACGGGGATGTAAAAGC	This paper	N/A
*Hbb* qPCR forward primer: GCTGGTTGTCTACCCTTGGA	This paper	N/A
*Hbb* qPCR reverse primer: GGCCTTCACTTTGGCATTAC	This paper	N/A
*Eif1a* qPCR forward primer: AAGCTCTTCCTGGGGACAAT	This paper	N/A
*Eif1a* qPCR reverse primer: ATGCTATGTGGGCTGTGTGA	This paper	N/A
*Gapdh* qPCR forward primer: CCGTAGACAAAATGGTGAAGG	This paper	N/A
*Gapdh* qPCR reverse primer: CAATCTCCACTTTGCCACTGC	This paper	N/A

Recombinant DNA		

U6-stuffer-longTracer-GFP lentivirus vector	This paper	N/A
psPAX2 packaging vector	This paper	Addgene Cat# 12260
pmD2.G envelope vector	This paper	Addgene Cat# 12259

Software and Algorithms		

ImageJ	Schneider et al., 2012^52^	https://imagej.nih.gov/ij/
GraphPad Prism	https://www.graphpad.com/scientific-software/prism/	v7.01
ChIP sequencing reads	NCBI’s GEO under accession number GSE248876	(https://www.ncbi.nlm.nih.gov/geo/query/acc.cgi?acc=GSE248876)
ChIP-seq code availability	This Paper	https://github.com/sanderslab/five_tr_chip

## Highlights

- ASD-associated TRs share substantial genomic binding sites in developing human and mouse cortex
- The overlap within a promoter region was highly predictive of brain-expressed genes
- Functional convergence of ASD-associated TRs leads to shared neurodevelopmental disorders
