# Supplementary material for: Five autism-associated transcriptional regulators target shared loci proximal to brain-expressed genes
Source: Cell Rep. Author manuscript; Available in PMC 2024 Jul 10. (PMC11235582; doi:10.1016/j.celrep.2024.114329)
Supplement: 1 [file NIHMS2005245-supplement-1.pdf]

Cell Reports, Volume *43*

Supplemental information

Five autism-associated transcriptional regulators target shared loci proximal to brain-expressed genes

Siavash Fazel Darbandi, Joon-Yong An, Kenneth Lim, Nicholas F. Page, Lindsay Liang, David M. Young, Athena R. Ypsilanti, Matthew W. State, Alex S. Nord, Stephan J. Sanders, and John L.R. Rubenstein

SUPPLEMENTAL FIGURES:

Figure S1. Related to figure 1. Read counts across all peaks in human and mouse. A) The analysis in Fig. 1D showing read counts with 1kbp around the middle of ChIP-seq peaks in human cortex but also including all peaks from ATAC-seq in human cortex performed at GW18 and GW19. The peaks are shown in the same order on the y-axis for all eight datasets, including inputs and blocking peptides (BP) as negative controls. **B)** Read counts arranged by peak count are shown for all replicates, inputs, and blocking peptides of ChIP-seq performed on mouse cortex at E15.5. Again, the peaks are shown in the same order on the y-axis for all datasets shown. **C)** The analysis in 'B' is repeated for all samples at E18.5 in mouse cortex.

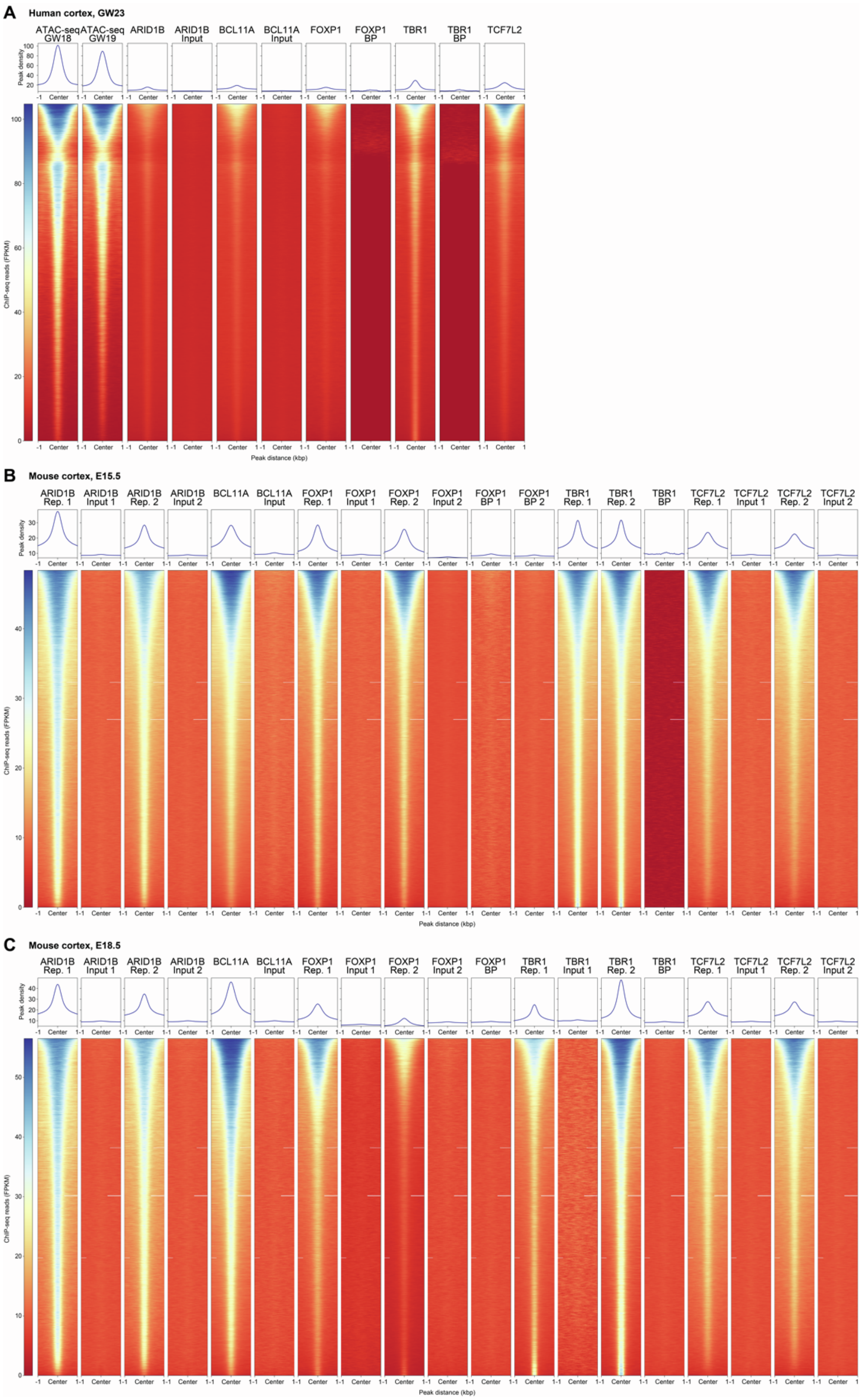
A
Human cortex, GW23
ATAC-seq GW18
ATAC-seq GW19
ARID1B
ARID1B Input
BCL11A
BCL11A Input
FOXP1
FOXP1 BP
TBR1
TBR1 BP
TCF7L2
Peak density
ChIP-seq reads (FPKM)
Center
Peak distance (kbp)
B
Mouse cortex, E15.5
ARID1B Rep. 1
ARID1B Input 1
ARID1B Rep. 2
ARID1B Input 2
BCL11A
BCL11A Input
FOXP1 Rep. 1
FOXP1 Input 1
FOXP1 Rep. 2
FOXP1 Input 2
FOXP1 BP 1
FOXP1 BP 2
TBR1 Rep. 1
TBR1 Rep. 2
TBR1 BP
TCF7L2 Rep. 1
TCF7L2 Input 1
TCF7L2 Rep. 2
TCF7L2 Input 2
C
Mouse cortex, E18.5
ARID1B Rep. 1
ARID1B Input 1
ARID1B Rep. 2
ARID1B Input 2
BCL11A
BCL11A Input
FOXP1 Rep. 1
FOXP1 Input 1
FOXP1 Rep. 2
FOXP1 Input 2
FOXP1 BP
TBR1 Rep. 1
TBR1 Input 1
TBR1 Rep. 2
TBR1 BP
TCF7L2 Rep. 1
TCF7L2 Input 1
TCF7L2 Rep. 2
TCF7L2 Input 2

Figure S2. Related to figure 1. Reproducibility of peaks across replicates in mouse cortex. A) Two ChIP-seq replicates (replicate 1 in blue and replicate 2 in red) were performed for the four TRs (ARID1B, FOXP1, TBR1, and TCF7L2) at two developmental stages (E15.5 and E18.5). The number of nucleotides in each replicate is shown on the y-axis with darker colors showing nucleotides overlapping between replicates and lighter colors showing nucleotides unique to one replicate. **B)** The analysis in 'A' is repeated but with nucleotide count replaced by the number of peaks on the y-axis. The number of overlapping peaks (defined as any overlap) varies between replicates because one peak in replicate 1 may overlap multiple peaks in replicate 2 and *vice versa*. **C)** Using the same methods as 'A', TBR1 E15.5 Replicates 1 and 2 from this manuscript are compared to two biological replicates (Notwell R1 and Notwell R2) of TBR1 E15.5 mouse cortex data published previously (*13*). The percent of overlapping nucleotides is shown for the Notwell replicates. **D)** The analysis in 'C' is repeated but using overlapping peaks instead of overlapping nucleotides. **(E) Enrichment of TR motif in ChIP-seq data.** Motifs were identified from Homer for BCL11A, FOXP1, TBR1, and TCF7L2; no equivalent motif was available for ARID1B. Using Homer, the proportion of peaks with the corresponding motif (e.g., BCL11A motif in BCL11A ChIP-seq peaks) was compared to the proportion of sequence scrambled peaks with the corresponding motif to generate a relative risk (y-axis). Error bars represent the 95% confidence interval calculated from the log-transformed risk ratio; P-values are calculated by Homer using the binomial exact test.

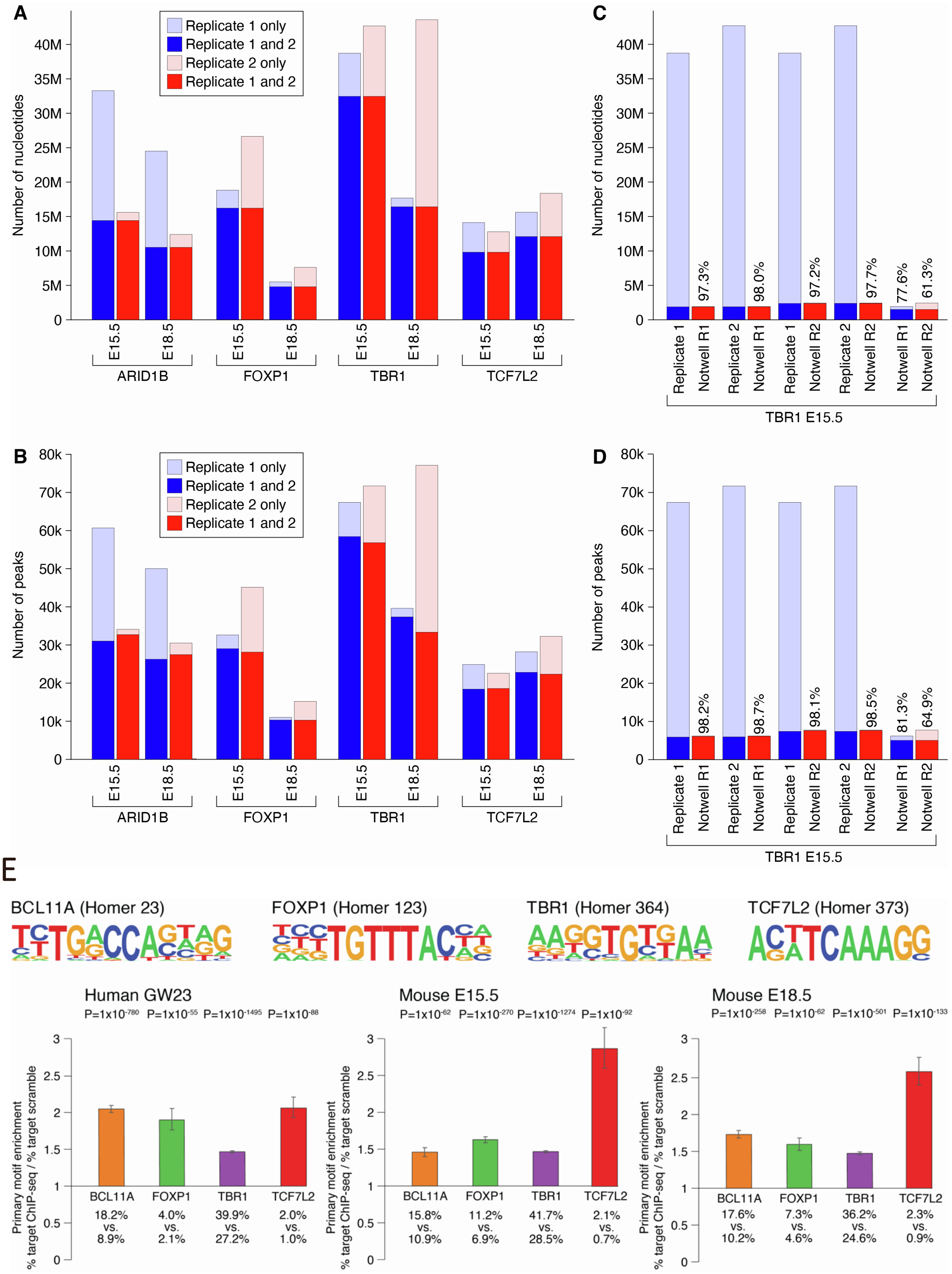

A
Replicate 1 only
Replicate 1 and 2
Replicate 2 only
Replicate 1 and 2
Number of nucleotides
0
5M
10M
15M
20M
25M
30M
35M
40M
E15.5
E18.5
ARID1B
FOXP1
TBR1
TCF7L2
C
97.3%
98.0%
97.2%
97.7%
77.6%
61.3%
Replicate 1
Notwell R1
Replicate 2
Notwell R1
Replicate 1
Notwell R2
Replicate 2
Notwell R2
Notwell R1
Notwell R2
TBR1 E15.5
B
Number of peaks
0
10k
20k
30k
40k
50k
60k
70k
80k
D
98.2%
98.7%
98.1%
98.5%
81.3%
64.9%
E
BCL11A (Homer 23)
FOXP1 (Homer 123)
TBR1 (Homer 364)
TCF7L2 (Homer 373)
Human GW23
$P=1x10^{-780}$ $P=1x10^{-55}$ $P=1x10^{-1495}$ $P=1x10^{-88}$
Mouse E15.5
$P=1x10^{-62}$ $P=1x10^{-270}$ $P=1x10^{-1274}$ $P=1x10^{-92}$
Mouse E18.5
$P=1x10^{-258}$ $P=1x10^{-62}$ $P=1x10^{-501}$ $P=1x10^{-133}$
Primary motif enrichment
% target ChIP-seq / % target scramble
BCL11A
FOXP1
TBR1
TCF7L2
18.2% vs. 8.9%
4.0% vs. 2.1%
39.9% vs. 27.2%
2.0% vs. 1.0%
15.8% vs. 10.9%
11.2% vs. 6.9%
41.7% vs. 28.5%
2.1% vs. 0.7%
17.6% vs. 10.2%
7.3% vs. 4.6%
36.2% vs. 24.6%
2.3% vs. 0.9%

Figure S3. Related to figure 2. Overlap between TRs and epigenetic markers. A) Top 10,000 proximal ChIP-seq peaks ranked by p-value from 14 TRs in adult human liver (ENCODE) and five ASD-associated TRs in fetal human cortex at GW23 to assess the proportion of peaks that intersect. B) Equivalent plot for 8,000 distal peaks. C) Overlap between proximal ChIP-seq peaks for the five ASD-associated TRs in developing human cortex overlapping with H3K27ac ChIP-seq peaks. Color gradient represents the percentage of peaks in each section with red being the highest percentage and white being 0%; peak counts shown in each section. D) Equivalent plot for proximal peaks that do not overlap with H3K27ac ChIP-seq peaks, E) distal peaks that do overlap with H3K27ac ChIP-seq peaks, and F) distal peaks that do not overlap with H3K27ac ChIP-seq peaks. G) For each ASD-associated TR, ChIP-seq proximal peaks are ranked by p-value (with 1 being the highest confidence) and split between 5TRa peaks (left, purple) and all other TR peaks (right, green). H) Equivalent plot to 'G' for distal peaks. Abbreviations: 5TRa: peak with all five ASD-associated transcription factors (5TR) and ATAC-seq (a); GW23: gestational week 23. Statistical analyses: A and B: Wilcoxon test.

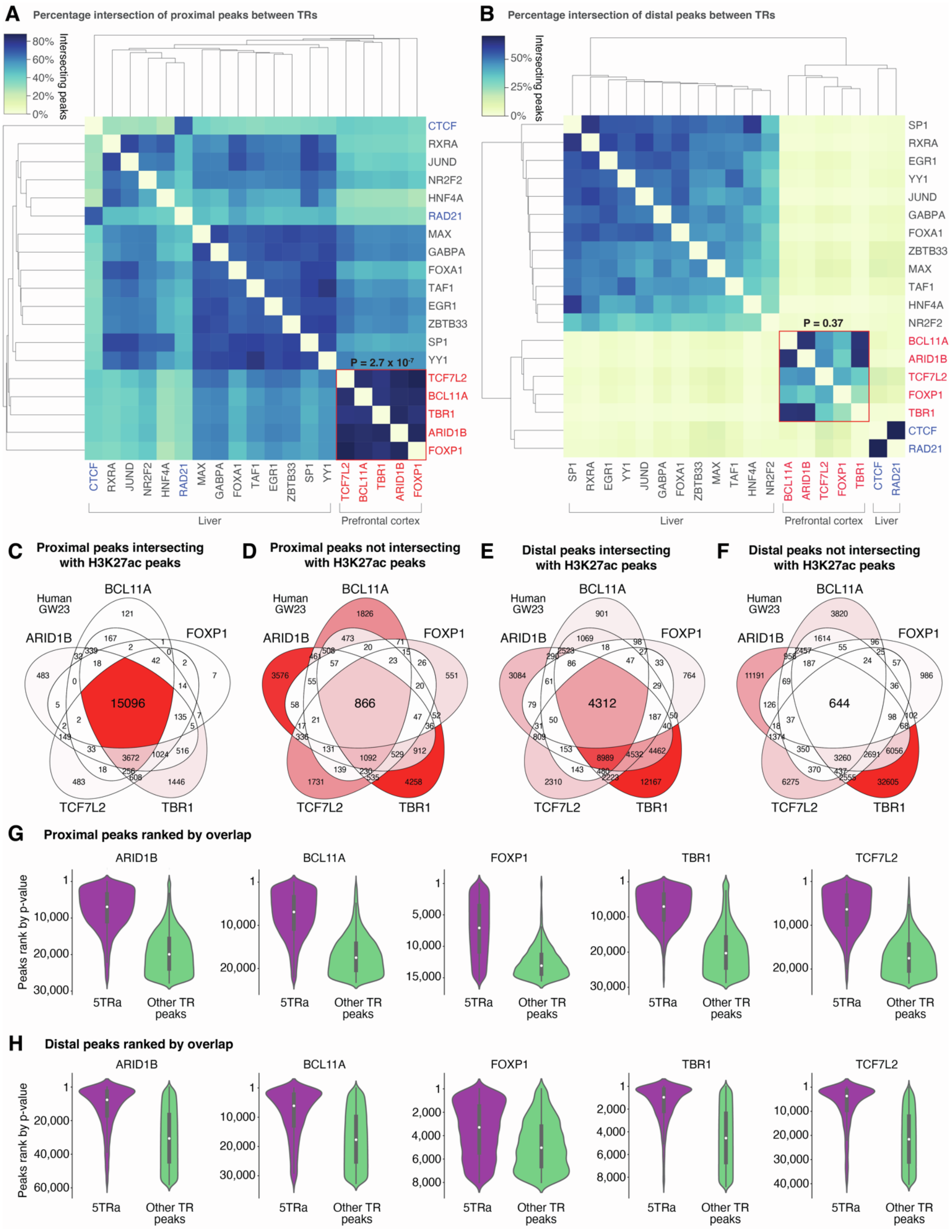
A Percentage intersection of proximal peaks between TRs
Intersecting peaks
80%
60%
40%
20%
0%
CTCF
RXRA
JUND
NR2F2
HNF4A
RAD21
MAX
GABPA
FOXA1
TAF1
EGR1
ZBTB33
SP1
YY1
TCF7L2
BCL11A
TBR1
ARID1B
FOXP1
P = 2.7 x 10-7
Liver
Prefrontal cortex
B Percentage intersection of distal peaks between TRs
Intersecting peaks
50%
25%
0%
SP1
RXRA
EGR1
YY1
JUND
GABPA
FOXA1
ZBTB33
MAX
TAF1
HNF4A
NR2F2
BCL11A
ARID1B
TCF7L2
FOXP1
TBR1
CTCF
RAD21
P = 0.37
Liver
Prefrontal cortex
Liver
C Proximal peaks intersecting with H3K27ac peaks
D Proximal peaks not intersecting with H3K27ac peaks
E Distal peaks intersecting with H3K27ac peaks
F Distal peaks not intersecting with H3K27ac peaks
Human GW23
BCL11A
ARID1B
FOXP1
TCF7L2
TBR1
15096
866
4312
644
G Proximal peaks ranked by overlap
H Distal peaks ranked by overlap
ARID1B
BCL11A
FOXP1
TBR1
TCF7L2
Peaks rank by p-value
5TRa
Other TR peaks

Figure S4. Related to figure 2. Permutation analysis of overlapping peaks. A) Left: Overlap of proximal peaks from each of the five ASD-associated TRs in GW23 human cortex was assessed and plotted as a Venn diagram. Intensity of red indicates the proportion of the total unique loci that are within that section, scaled from 0% (white) to the maximum (41.2%, red). **Middle left:** The expected number of overlaps is estimated by permutation analysis. For each permutation, the peaks of all five TRs are concatenated to create the search space, then, for each of the five TRs in turn, the same number of peaks as the original data are selected at random from the concatenated peak list. This is repeated for each of the five TRs then the overlap between permuted TRs is assessed. This process was repeated 1,000 times. The mean counts are shown in each section of the Venn diagram, with intensity of red scaled from 0% (white) to the maximum (22.1%, red). **Middle right:** The difference between the observed and mean permuted expectation and the standard deviation of the permutations is used to estimate the effect size (Cohen's *d*) in each section. A positive Cohen's *d* (more peaks in observed than expected) is shown in red, a Cohen's *d* of 0 is white, while a negative Cohen's *d* (fewer peaks in observed than expected) is shown in blue. Intensity of color represents a linear scale of Cohen's *d* between -0.6 and 0.6. **Right:** The distribution of expected counts for the overlap of all five TRs (5TR) is shown as a histogram on a logarithmic axis with the observed count shown as a red line. The standard deviation is used to estimate the z-score, which is converted to a P-value. **B)** The methodology described in 'A' is repeated for proximal (left) and distal (right) peaks, split by whether the peaks overlap with peaks from GW18/19 human cortex ATAC-seq. The effect size Venn diagram and histogram of expected vs. observed overlap of all five TRs are shown. **C)** The analysis in 'B' is repeated for E15.5 mouse cortex ChIP-seq and E15.5/E18.5 mouse cortex ATAC-seq data. **D)** The analysis in 'B' is repeated for E18.5 mouse cortex ChIP-seq and E15.5/E18.5 mouse cortex ATAC-seq data.

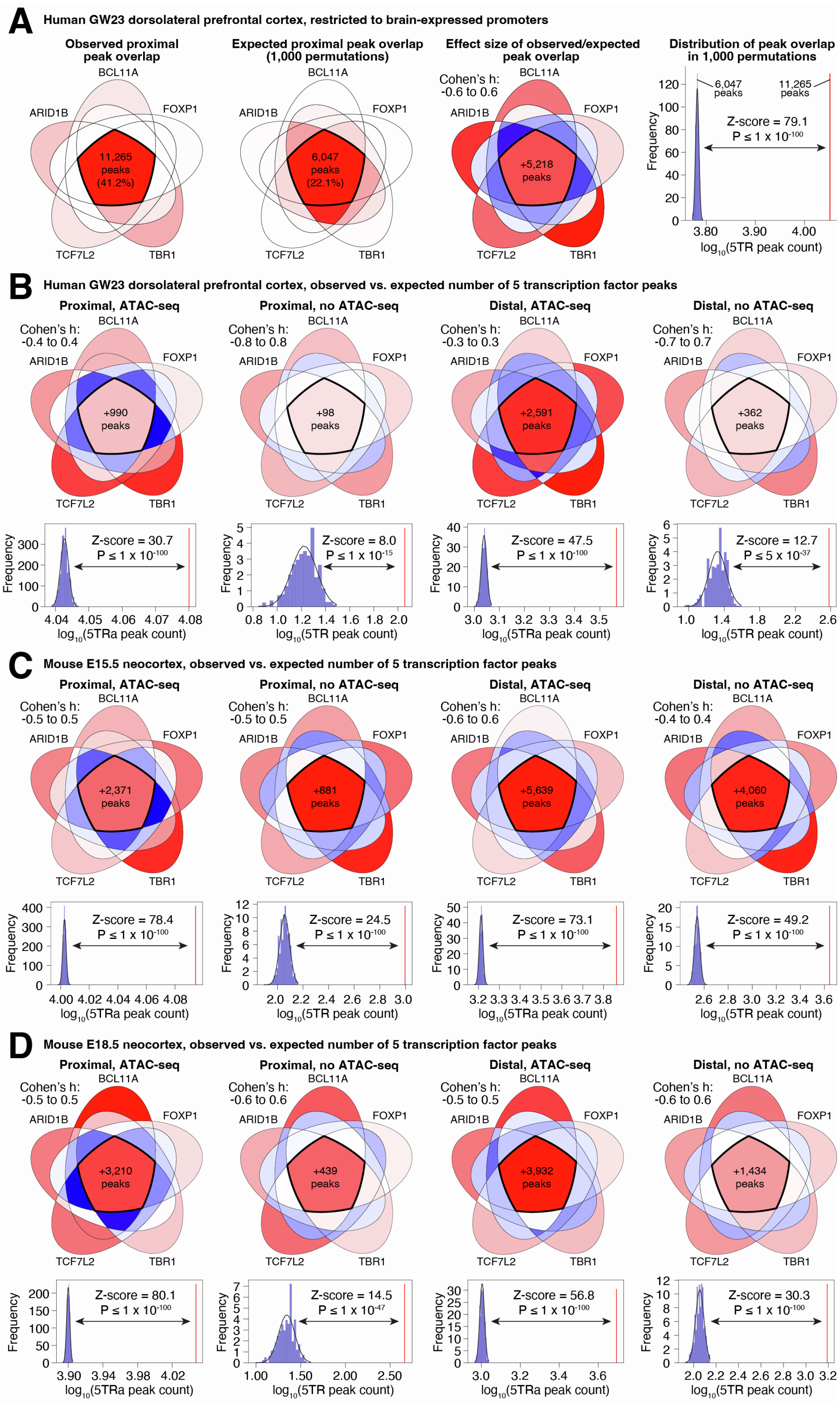
A
Human GW23 dorsolateral prefrontal cortex, restricted to brain-expressed promoters
Observed proximal peak overlap
Expected proximal peak overlap (1,000 permutations)
Effect size of observed/expected peak overlap
Distribution of peak overlap in 1,000 permutations
BCL11A
ARID1B
FOXP1
TCF7L2
TBR1
11,265 peaks (41.2%)
6,047 peaks (22.1%)
Cohen's h: -0.6 to 0.6
+5,218 peaks
6,047 peaks
11,265 peaks
Z-score = 79.1
P ≤ 1 x 10-100
Frequency
log10(5TR peak count)
B
Human GW23 dorsolateral prefrontal cortex, observed vs. expected number of 5 transcription factor peaks
Proximal, ATAC-seq
Proximal, no ATAC-seq
Distal, ATAC-seq
Distal, no ATAC-seq
Cohen's h: -0.4 to 0.4
+990 peaks
Cohen's h: -0.8 to 0.8
+98 peaks
Cohen's h: -0.3 to 0.3
+2,591 peaks
Cohen's h: -0.7 to 0.7
+362 peaks
Z-score = 30.7
P ≤ 1 x 10-100
Z-score = 8.0
P ≤ 1 x 10-15
Z-score = 47.5
P ≤ 1 x 10-100
Z-score = 12.7
P ≤ 5 x 10-37
log10(5TRa peak count)
log10(5TR peak count)
C
Mouse E15.5 neocortex, observed vs. expected number of 5 transcription factor peaks
Cohen's h: -0.5 to 0.5
+2,371 peaks
Cohen's h: -0.5 to 0.5
+881 peaks
Cohen's h: -0.6 to 0.6
+5,639 peaks
Cohen's h: -0.4 to 0.4
+4,060 peaks
Z-score = 78.4
P ≤ 1 x 10-100
Z-score = 24.5
P ≤ 1 x 10-100
Z-score = 73.1
P ≤ 1 x 10-100
Z-score = 49.2
P ≤ 1 x 10-100
D
Mouse E18.5 neocortex, observed vs. expected number of 5 transcription factor peaks
Cohen's h: -0.5 to 0.5
+3,210 peaks
Cohen's h: -0.6 to 0.6
+439 peaks
Cohen's h: -0.5 to 0.5
+3,932 peaks
Cohen's h: -0.6 to 0.6
+1,434 peaks
Z-score = 80.1
P ≤ 1 x 10-100
Z-score = 14.5
P ≤ 1 x 10-47
Z-score = 56.8
P ≤ 1 x 10-100
Z-score = 30.3
P ≤ 1 x 10-100

Figure S5. Related to figure 2. Peak overlap with increasing p-value thresholds. The analysis in Figure 2E/F is repeated with the ChIP-seq peaks filtered by P-value threshold. The top line shows a threshold of $P \leq 0.0013$, as used in the primary analysis. The middle line only includes peaks at a threshold of $P \leq 1 \times 10^{-10}$, while the bottom line only includes peaks at a threshold of $P \leq 1 \times 10^{-15}$. ATAC-seq peaks were included with a threshold of $P \leq 0.0013$ throughout.

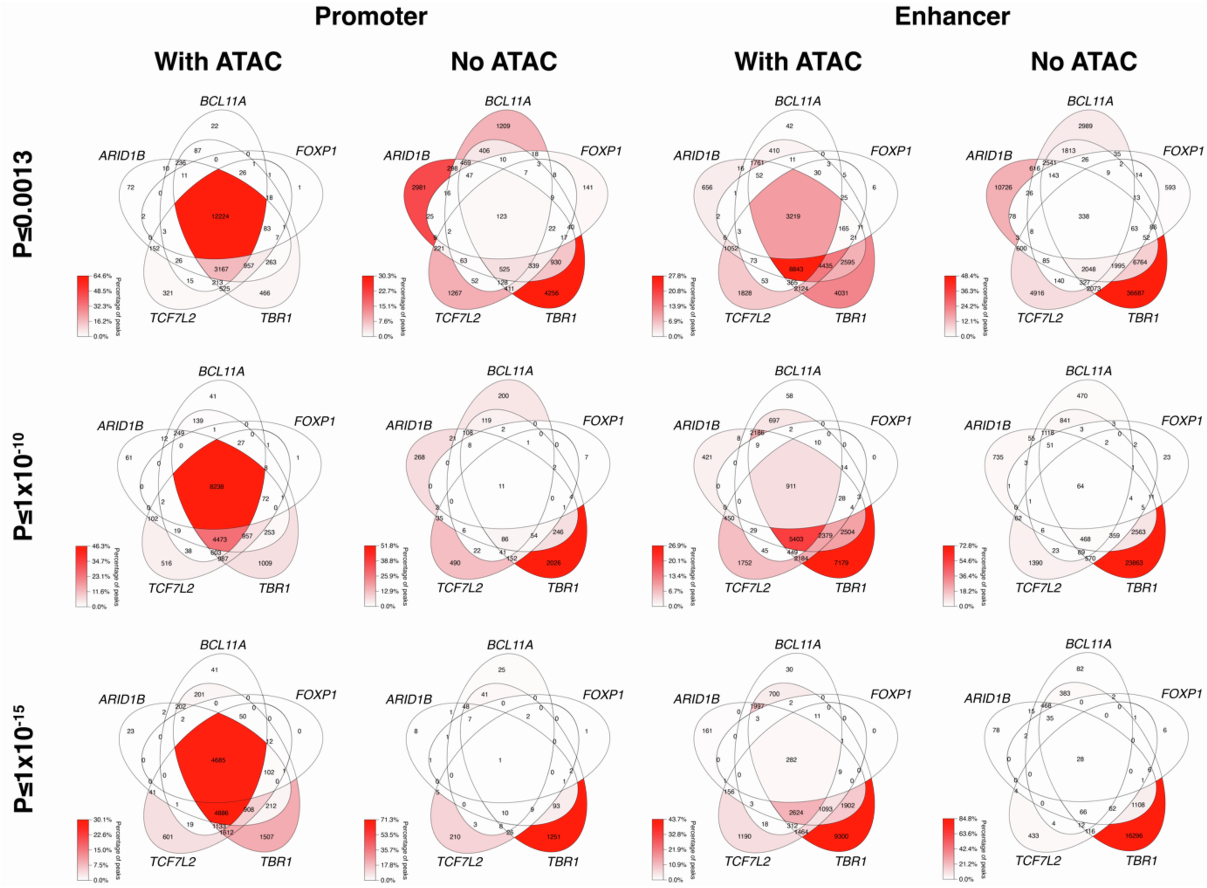

Figure S6. Related to figure 4. (i) Relationship between peak significance at the promoter and gene expression in the midfetal brain. Dots are colored by peak density, with navy representing a single peak and yellow representing multiple peaks (~20). All genes are shown on the same axes for comparison. The red line represents the lowess curve. **(ii) Overlap between ≥3TRa peaks and validated vista enhancers. (A)** The odds of VISTA enhancer activity in a given tissue based on the presence of a distal ≥3TRa peak. ***FDR<0.001, ****FDR<0.0001. **(B)** The analysis in 'A' is repeated for a subset of VISTA enhancer sequences reclassified by expression patterns in the pallium and subpallium (Table S6). **(iii) Longitudinal assessment of changes in *Arid1b* and *Tbr1* expression *in vitro* following CRISPRi treatment**. CRISPRi guides were designed against promoter regions of *Arid1b* and *Tbr1.* Changes in *Arid1b* and *Tbr1* expression levels were compared to scrambled guides and the *Ef1a* housekeeping gene. CRISPRi reduced relative expression of *Arid1b* to 75% by day 2 and 25% by day 8; likewise, *Tbr1* was reduced to 50% by day 2 and 5% by day 8.

(i)

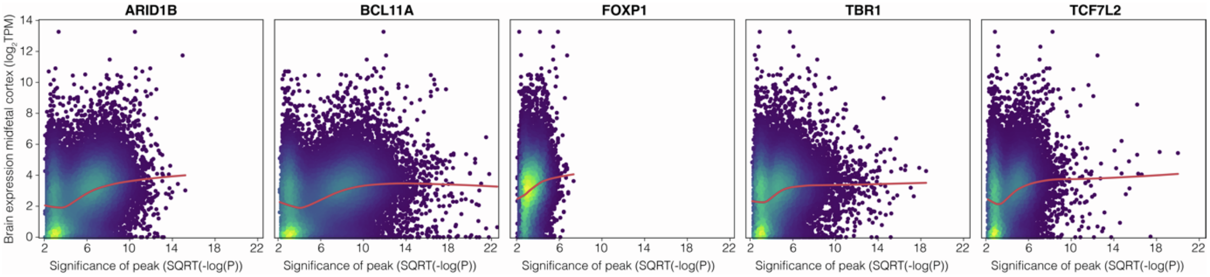

(ii)

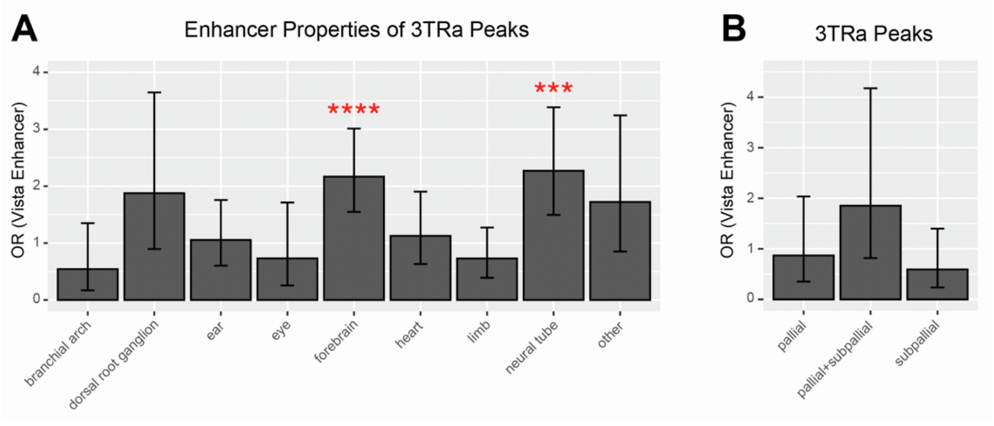

(iii)

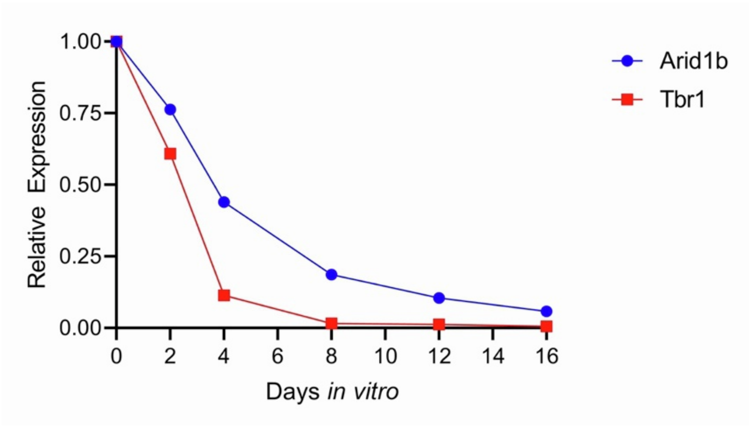

Figure S7. Related to figure 6. Cell type enrichment and functional consequences. A) Expression of the five TRs in human prenatal cortex by cell type cluster (Herring et al. PMID: 36318921). **B)** Cell type clusters from the human fetal cortex (*17*) were assessed for enrichment of genes targeted by all five ASD-associated TRs and ATAC-seq (5TRa) in human (GW23) fetal cortex. The degree of enrichment is indicated by color; significance threshold is indicated by box size/asterisk. **C)** Cell type proportions by major classes of cell types, as estimated by deconvolution of bulk RNA-seq in postmortem brain from ASD cases and controls (*8*).

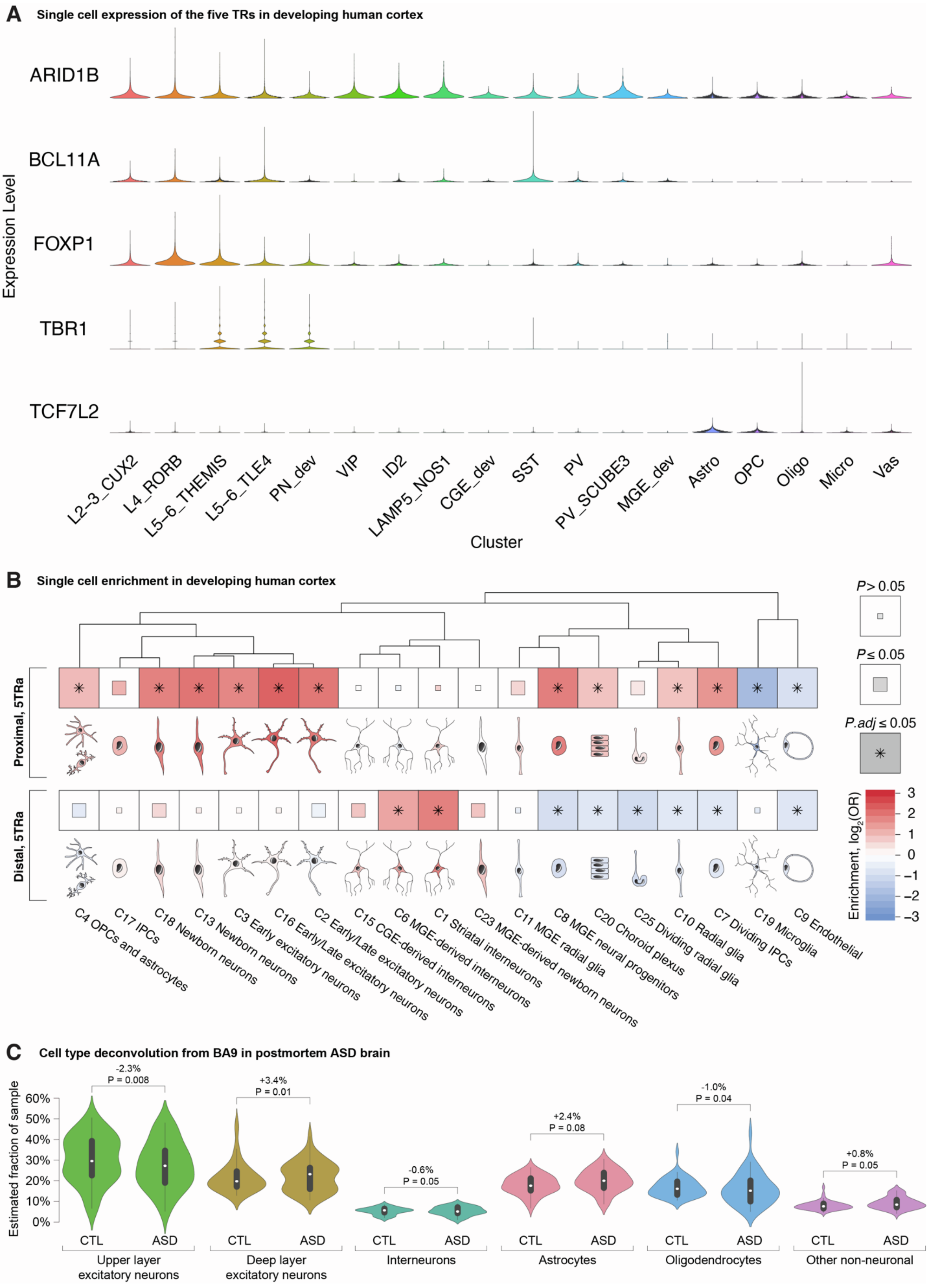
A Single cell expression of the five TRs in developing human cortex
ARID1B
BCL11A
FOXP1
TBR1
TCF7L2
Expression Level
L2–3_CUX2
L4_RORB
L5–6_THEMIS
L5–6_TLE4
PN_dev
VIP
ID2
LAMP5_NOS1
CGE_dev
SST
PV
PV_SCUBE3
MGE_dev
Astro
OPC
Oligo
Micro
Vas
Cluster
B Single cell enrichment in developing human cortex
Proximal, 5TRa
Distal, 5TRa
P > 0.05
P ≤ 0.05
P.adj ≤ 0.05
Enrichment, log2(OR)
3
2
1
0
−1
−2
−3
C4 OPCs and astrocytes
C17 IPCs
C18 Newborn neurons
C13 Newborn neurons
C3 Early excitatory neurons
C16 Early/Late excitatory neurons
C2 Early/Late excitatory neurons
C15 CGE-derived interneurons
C6 MGE-derived interneurons
C1 Striatal interneurons
C23 MGE-derived newborn neurons
C11 MGE radial glia
C8 MGE neural progenitors
C20 Choroid plexus
C25 Dividing radial glia
C10 Radial glia
C7 Dividing IPCs
C19 Microglia
C9 Endothelial
C Cell type deconvolution from BA9 in postmortem ASD brain
Estimated fraction of sample
60%
50%
40%
30%
20%
10%
0%
-2.3%
P = 0.008
+3.4%
P = 0.01
-0.6%
P = 0.05
+2.4%
P = 0.08
-1.0%
P = 0.04
+0.8%
P = 0.05
CTL
ASD
Upper layer excitatory neurons
Deep layer excitatory neurons
Interneurons
Astrocytes
Oligodendrocytes
Other non-neuronal
